# Structural Investigations of Human A2M Identify a Hollow Native Conformation That Underlies Its Distinctive Protease-Trapping Mechanism

**DOI:** 10.1016/j.mcpro.2021.100090

**Published:** 2021-05-06

**Authors:** Seandean Lykke Harwood, Jeppe Lyngsø, Alessandra Zarantonello, Katarzyna Kjøge, Peter Kresten Nielsen, Gregers Rom Andersen, Jan Skov Pedersen, Jan J. Enghild

**Affiliations:** 1 Department of Molecular Biology and Genetics, Aarhus University, Aarhus, Denmark; 2 Global Research Technologies, Novo Nordisk A/S, Måløv, Denmark; 3 Interdisciplinary Nanoscience Center, Aarhus University, Aarhus, Denmark; 4 Department of Chemistry, Aarhus University, Aarhus, Denmark

**Keywords:** alpha 2 macroglobulin, alpha-macroglobulins, macroglobulins, protease inhibitor, protein conformation, structural model, conformational change, protein cross-linking, small-angle X-ray scattering, electron microscopy, α1-i3, alpha-1 inhibitor 3, a monomeric rat protease inhibitor, αM, alpha-macroglobulin superfamily, A2M^3K^, recombinant A2M with the Arg704Lys, Arg715Lys, and Arg719Lys mutations, A2M^LNK/LNK^, recombinant A2M with the Thr654Cys and Thr661Cys mutations, A2M, α_2_-macroglobulin (human, if not otherwise specified), A2M-MA, A2M treated with methylamine, A2M-T, A2M which has been cleaved by trypsin, CUB, the complement subcomponent C1r/C1s, urchin embryonic growth factor and bone morphogenetic protein 1 domain, DSSO, disuccinimidyl sulfoxide, ECAM, *E. coli* α_2_-macroglobulin, EM, electron microscopy, HBS, HEPES-buffered saline, here defined as 20 mm HEPES-NaOH, 150 mM NaCl, pH 7.4, IFT, indirect Fourier transform, LNK, linker region, LRP1, low-density lipoprotein receptor-related protein 1, MA, methylamine, MG, macroglobulin domain, PMSF, phenylmethanesulfonyl fluoride, RB, receptor-binding domain;corresponds to MG8 in complement factors, SAXS, small-angle X-ray scattering, SEC, size-exclusion chromatography, TE, thiol ester domain, XL-MS, cross-linking-mass spectrometry

## Abstract

Human α_2_-macroglobulin (A2M) is the most characterized protease inhibitor in the alpha-macroglobulin (αM) superfamily, but the structure of its native conformation has not been determined. Here, we combined negative stain electron microscopy (EM), small-angle X-ray scattering (SAXS), and cross-linking–mass spectrometry (XL-MS) to investigate native A2M and its collapsed conformations that are obtained through aminolysis of its thiol ester by methylamine or cleavage of its bait region by trypsin. The combined interpretation of these data resulted in a model of the native A2M tetramer and its conformational changes. Native A2M consists of two crescent-shaped disulfide-bridged subunit dimers, which face toward each other and surround a central hollow space. In native A2M, interactions across the disulfide-bridged dimers are minimal, with a single major interface between the linker (LNK) regions of oppositely positioned subunits. Bait region cleavage induces both intrasubunit domain repositioning and an altered configuration of the disulfide-bridged dimer. These changes collapse the tetramer into a more compact conformation, which encloses an interior protease-trapping cavity. A recombinant A2M with a modified bait region was used to map the bait region’s position in native A2M by XL-MS. A second recombinant A2M introduced an intersubunit disulfide into the LNK region, demonstrating the predicted interactions between these regions in native A2M. Altogether, our native A2M model provides a structural foundation for understanding A2M’s protease-trapping mechanism, its conformation-dependent receptor interactions, and the dissociation of native A2M into dimers due to inflammatory oxidative stress.

## Highlights

• Native A2M is hollow and tube-like.
• A2M’s bait regions are oriented inward and are accessed from inside A2M.
• A2M tetramerizes through symmetrical interactions between its LNK regions.
• The receptor-binding site is in an inaccessible position inside native A2M.

## Graphical Abstract

**Figure:**
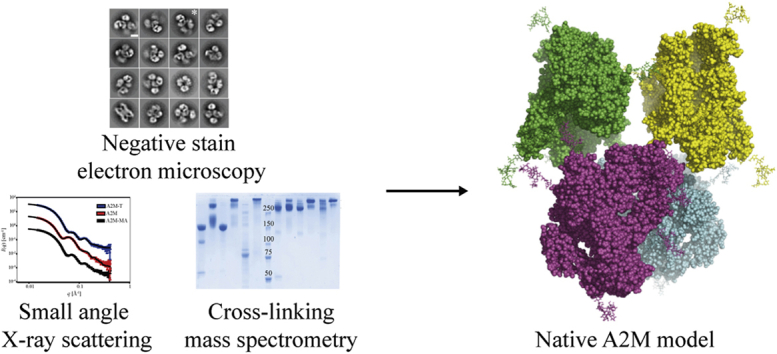

## In Brief

The native conformation of the protease inhibitor A2M was investigated using negative stain electron microscopy, small-angle X-ray scattering, and cross-linking mass spectrometry. The low-resolution model built from these data describes a hollow tubular configuration that explains several aspects of A2M’s unique trapping mechanism. This model was further validated by two recombinantly expressed A2M mutants, which probed the location of the bait region and demonstrated the existence of a critical interface between A2M’s disulfide-bridged dimers.

Human α_2_-macroglobulin (A2M), a 720 kDa homotetramer present at ~1 to 2 g per liter in plasma (1), is the eponymous and archetypical protease inhibitor of the αM superfamily. A2M has a unique mechanism of protease inhibition that is representative of αM protease inhibitors in general. Furthermore, several characteristics of the αM protease inhibitors are conserved in the αM complement components C3 and C4, including their reactive thiol ester moiety and a proteolysis-induced conformational change, which regulates binding to other proteins.

Protease inhibition by A2M and other αMs is enacted by a unique trapping mechanism whereby proteases are sequestered inside the αM protease inhibitor, restricting their access to protein substrates (2). αM protease inhibitors have a bait region that is highly susceptible to proteolysis (3), and its cleavage triggers a dramatic structural collapse (4, 5, 6, 7, 8, 9), during which the instigating protease becomes trapped (10). The ability of a protease to cleave the bait region is the only known factor determining whether an αM will inhibit a protease. As αMs do not interact directly with proteases’ active sites, they can inhibit proteases of all classes.

Most αM proteins have a thiol ester group formed between the cysteine and glutamine residues of a conserved CGEQ motif located in the thiol ester (TE) domain. In native αM proteins, the thiol ester is protected in a hydrophobic environment formed by the TE, macroglobulin (MG) 2, and MG8 domains, as seen in structures of C3 and C4 (11, 12, 13); MG8 corresponds to the receptor binding (RB) domain of A2M. Following the conformational changes induced by proteolytic activation, the thiol ester becomes exposed and can be attacked by nucleophiles. The thiol esters of αM protease inhibitors are used to conjugate proteases, predominantly through their primary amine groups (14). This covalent conjugation is required for protease inhibition by monomeric inhibitors such as human A2M-like protein 1, rat α1-i3, and *Escherichia coli* ECAM (15, 16, 17), but not by tetrameric αMs such as A2M and ovostatin (18, 19). Some small nucleophiles, such as methylamine, can access and react with the thiol ester of A2M in its native conformation (10). This triggers a conformational change in human A2M to a collapsed conformation similar to the proteolysis-induced conformation (7, 10, 20).

αM proteins consist of a ~180 kDa base unit, which may constitute a functional monomer, as is the case for complement C3 and C4, or may assemble into homodimers or homotetramers such as A2M. Crystal structures of complement components C3 and C4 and their proteolytically activated forms C3b and C4b defined a mostly conserved structure of the αM base unit. This base unit contains a static structure called the MG ring comprising MG domains 1 to 6 and several dynamic domains, most notably the TE domain, which are repositioned during the proteolysis-induced conformational change (11, 12, 13, 21, 22). For the tetrameric αM protease inhibitors, persistent efforts have produced a crystal structure of methylamine-treated A2M (A2M-MA) in which the A2M-MA tetramer exhibits approximate D_2_ symmetry (23). This confirmed the C3b-like structure of the A2M-MA subunit expected due to the high degree of shared sequence identity between A2M and C3 (24, 25). The crystal structure also supported the previously determined organization of the A2M tetramer as a dimer of disulfide-bridged dimers (26) and showed how the collapsed A2M tetramer encloses two protease-trapping chambers. There is no high-resolution structure of native A2M, but ~25 Å resolution maps have been constructed using EM (4, 9). These EM maps of native A2M were produced before the overall αM architecture had been identified, and no modeling of A2M’s domains or subunits was performed.

This study investigated the native conformation of A2M using EM, SAXS, and XL-MS. A2M-MA and trypsin-cleaved A2M (A2M-T) were included in the SAXS and XL-MS experiments for comparison. The resulting model of native A2M was challenged using two A2M mutants, A2M^3K^ and A2M^LNK/LNK^, which confirmed that the bait region is located in the A2M interior and that the LNK regions participate in intersubunit interactions in native A2M. We present a model of native A2M based on these results, which shows how the tetramer is held together and how proteases initially encounter the bait region.

## Experimental Procedures

### Experimental Design and Statistical Rationale

Three conformations of A2M were investigated by XL-MS: its native conformation directly following plasma purification, the chemically induced conformation obtained by methylamine treatment of the thiol ester, and the trypsin:A2M complex obtained by cleaving A2M’s bait region with trypsin. Furthermore, the monomer, dimer, and tetramer bands from cross-linked native A2M were compared with each other. These protein samples were prepared and cross-linked identically in three separate experiments, as assessed by SDS-PAGE and native PAGE. In-gel digest in preparation for LC-MS was not replicated. Each sample from in-gel digest was analyzed by LC-MS with five technical replicates using data-dependent acquisition (DDA) and five technical replicates using a method targeting the cross-links identified by DDA from all samples.

### Preparation of Native A2M, A2M-MA, and A2M-T

A2M was purified from plasma from a healthy volunteer using established protocols (27, 28) and was never frozen. HEPES-buffered saline (HBS; 20 mM HEPES-NaOH, 150 mM NaCl, pH 7.4) was used as the running buffer for SEC and as the base buffer for all experiments, unless noted otherwise.

To prepare A2M-MA, purified A2M from plasma was treated with 0.5 M of methylamine, pH eight at 37 °C for 24 h, followed by desalting into HBS on a PD-10 column (GE Healthcare). To prepare A2M-T, bovine pancreatic trypsin (Sigma-Aldrich) was added to A2M at a 2.4:1 protease:A2M M ratio and incubated for 2 min at 37 °C. Trypsin was then inhibited by 2 mM phenylmethanesulfonyl fluoride (PMSF) at room temperature for 15 min. Excess trypsin and PMSF were removed by SEC on a Sephacryl S-300 HR column.

To prepare deglycosylated A2M, A2M was incubated overnight at room temperature with a 1:10 w/w ratio deglycosidase:A2M of bacterially expressed PNGase F, N-glycosidase F, Endo F2, and Endo F3 (29), which were then removed by SEC. Deglycosylated A2M-MA and A2M-T were prepared from deglycosylated native A2M as described above.

### Preparation of Recombinant A2M Mutants

The design and cloning of plasmids for mammalian expression of recombinant A2M are described in Supplemental Methods. All recombinant A2M mutants were expressed in transiently transfected HEK293 Freestyle cells. The cells were transfected by mixing linear 25k polyethyleneimine (Polysciences) and plasmid DNA in a 4:1 w/w ratio in Freestyle medium (Thermo Fisher Scientific), incubating for 10 min, and then slowly dripping the mixture into a culture of cells at 1 × 10^6^ cells/ml, adding 1 μg of DNA per ml cell culture. After 4 days, the supernatant was harvested by spinning down the cells and neutralized by the addition of pH 7.4 HEPES-NaOH to 50 mM prior to A2M purification by the same protocol used for plasma-purified A2M.

### DSSO Cross-linking, In-gel Digests, and MS

Cross-linking of A2M preparations was performed in 120 mM HEPES-NaOH, pH 7.4, 150 mM NaCl, 5% v/v DMSO using 0.42 μM A2M and 420 μM DSSO. Cross-linking proceeded for 1 h at room temperature and was quenched by adding Tris-HCl at pH 7.4 to 50 mM. After cross-linking, samples were purified by SEC on a Sephacryl S-300 HR column into HBS.

To prepare cross-linked peptides for mass spectrometry, an in-gel digest approach was used. Samples were run on SDS-PAGE and the relevant bands were excised, shrunk in acetonitrile, reduced using 10 mM DTT, alkylated using 30 mM iodoacetamide, and digested overnight with 1:40 w/w MS-grade trypsin (Thermo Fisher Scientific) in 50 mM ammonium bicarbonate, pH 8, at 37 °C. Peptides were extracted from the gel bands and deglycosylated using PNGase F (New England Biolabs) for 1 h at 37 °C. A portion of the tryptic peptides was further digested using 1:30 w/w bovine pancreatic α-chymotrypsin (Thermo Fisher Scientific) or endoproteinase GluC (New England Biolabs) for 3 h at 37 °C. Prior to MS analysis, peptides were purified using pipette tips packed with POROS 50 R2 C18 resin (PerSeptive Biosystems).

LC-MS^3^ peptide analysis was performed using online reverse-phase separation of peptides over a 15 cm column packed in-house with Reprosil-Pur 120 C18-AQ 3 μM resin (Dr Maisch GmbH) on an EASY-nLC 1200 (Thermo Fisher Scientific) and an Orbitrap Eclipse Tribrid mass spectrometer (Thermo Fisher Scientific) running an MS^2^_MS^3^ method (30). At the MS^2^ level, peptides were fragmented by 20% energy collision-induced dissociation (CID); if the characteristic mass difference of DSSO fragment doublets was detected, these fragments were isolated for MS^3^ using 35% energy CID.

### XL-MS Data Analysis

Cross-linked peptides were identified in the MS data files with Proteome Discoverer (version 2.4 for samples digested with trypsin and GluC or trypsin alone, and version 2.3 for samples digested with both trypsin and chymotrypsin) and the XlinkX node (version 2.4), using established processing and consensus workflows for the analysis of MS^2^_MS^3^ spectra with MS-cleavable cross-links (30, 31, 32). Precursor mass tolerance was set to 10 ppm, MS^2^ fragment mass tolerance was 20 ppm, and MS^3^ fragment mass tolerance was 0.5 Da. Search databases included only the relevant proteins (A2M and trypsin). The false discovery rate was set to 0.01 using the XlinkX Validator node with a simple false discovery rate-determining strategy. Three missed cleavages were allowed for tryptic samples and five missed cleavages were allowed for the combination digests. Cysteine carbamidomethylation was a fixed modification, and methionine oxidation and asparagine deamidation (to account for removed N-glycans) were included as variable modifications. A2M’s original N-terminal at position 24 and neo-N-termini in the bait region after tryptic cleavage (positions 705, 716, and 720) were included as possible cross-linking sites. Misassignment of dead-end hydrolyzed cross-links to cross-links involving a single lysine residue was observed and manually corrected. Cross-link identifications with less than three cross-link spectral matches were filtered out, as were cross-links involving the ambiguous peptide RKE (which occurs twice when A2M is cleaved using both trypsin and GluC). Where noted, label-free quantification of cross-links extracted ion count areas using Proteome Discoverer 2.4 compared the abundance of cross-links across different samples.

Traces of protease-cleaved A2M in the plasma-purified native A2M sample or unconverted native A2M remaining in the A2M-MA and A2M-T samples constituted a potential source of cross-links, which would not reflect the structure of the conformation onto which they were mapped. Seven cross-links were identified in both native and collapsed A2M, which agreed with one model and conflicted with the other. Label-free quantification was used to compare their abundance across samples (supplemental Fig. S6, *C* and *D*). Five cross-links were disregarded in native A2M and two cross-links were disregarded in A2M-MA and A2M-T due to their at least tenfold greater abundance in the other conformation. These cross-links are indicated in Supplemental Spreadsheet 1.

### SDS-PAGE and Pore-Limited Native Page

Native pore-limited PAGE was performed as previously described (33). Denaturing SDS-PAGE was performed using the discontinuous 2-amino-2-methyl-1,3-propanediol and glycine buffer system with homemade 5 to 15% acrylamide gradient gels (34). Where noted, samples were reduced by 25 mM DTT at 95 °C for 5 min.

### Negative Stain Electron Microscopy

The A2M sample at 78 μg/ml was applied to a glow-discharged carbon-coated copper GC400 grid for 10 s. After application, the sample was blotted and stained with 3 μl of 2% w/v uranyl formate. The stain was immediately removed from the grid two times (wash steps), while the third time it was left on for 1 min before blotting and air drying the grid. The micrographs were recorded automatically in Leginon on a Tecnai T12 G2 transmission electron microscope operating at 120 kV and equipped with a Tietz TemCam-F416 detector (TVIPS). The defocus range during data collection was from −0.7 to −1.7 μm, with 750 ms exposure and 67,000-fold magnification, yielding a pixel size of 3.15 Å. The first CTF crossover was between 18 and 20 Å. A total of 45,000 particles were picked by autopicking in RELION (35) after 2D model generation based on 389 manually picked particles. The coordinates were extracted after CTF correction with a box size of 154 pixels. The particles used for 3D classification were selected after reference-free 2D classification in RELION 3.0.7, using a mask of 280 Å (30,000 particles). The selected particles were used for 3D classification in three classes based on automated generation of the initial model in RELION with D_2_ symmetry imposed. After 3D classification, only one of the classes resulted in a map where the four subunits could be clearly identified. This map was used as reference in 3D refinement.

### Small-Angle X-ray Scattering Data Collection

SAXS measurements were performed on a Bruker AXS NanoStar, equipped with an Excillum Ga liquid metal jet X-ray source and an optimized geometry (36) with relatively long side-by-side multilayer parabolic mirrors and large pinholes, where the one in front of the sample is a scatterless octagonal pinhole made in-house (37, 38). The data were recorded by a VÅNTEC-2000 (Bruker AXS) area-sensitive detector. The buffers of the samples were measured and used as backgrounds, and conversion to absolute scale was done using the scattering of a pure water sample at 20 °C. All samples were measured for 1800 s at 20 °C at a single concentration. Additional parameters during SAXS measurement are given in supplemental Table S1. The intensities are displayed as a function of the modulus of the scattering vector, q=4πsin(θ)/λ, where the X-ray wavelength λ is 1.34 Å and 2θ is the scattering angle. The data were rebinned to be approximately equidistantly distributed on a logarithmic *q* scale. The data quality and useful range of *q* were determined by performing Guinier fits and indirect Fourier transformations (IFTs) (39, 40). Kratky plots were used to assess compactness (41). Molecular masses were calculated from the forward scattering *I*(*q* = 0) using measured protein concentrations and a standard value of 2.0 × 10^10^ cm/g for the excess scattering length per unit mass, Δ*ρ*_*m*_.

### Model Construction and SAXS Rigid-Body Refinement

The initial models of A2M-MA and A2M-T for rigid-body refinement to the SAXS data were based on the A2M-MA crystal structure 4ACQ and the trypsin crystal structure 1F0T. The initial model of native A2M was based on fitting to the EM reconstruction (which used an A2M subunit with native C3-like domain positions). A detailed description is given in Supplemental Methods.

SAXS rigid-body refinement was performed as described in Supplemental Methods. Rigid-body definitions are given in Supplemental Table S2.

## Results

### Negative Stain Electron Microscopy of Native A2M

**Fig. 1:**
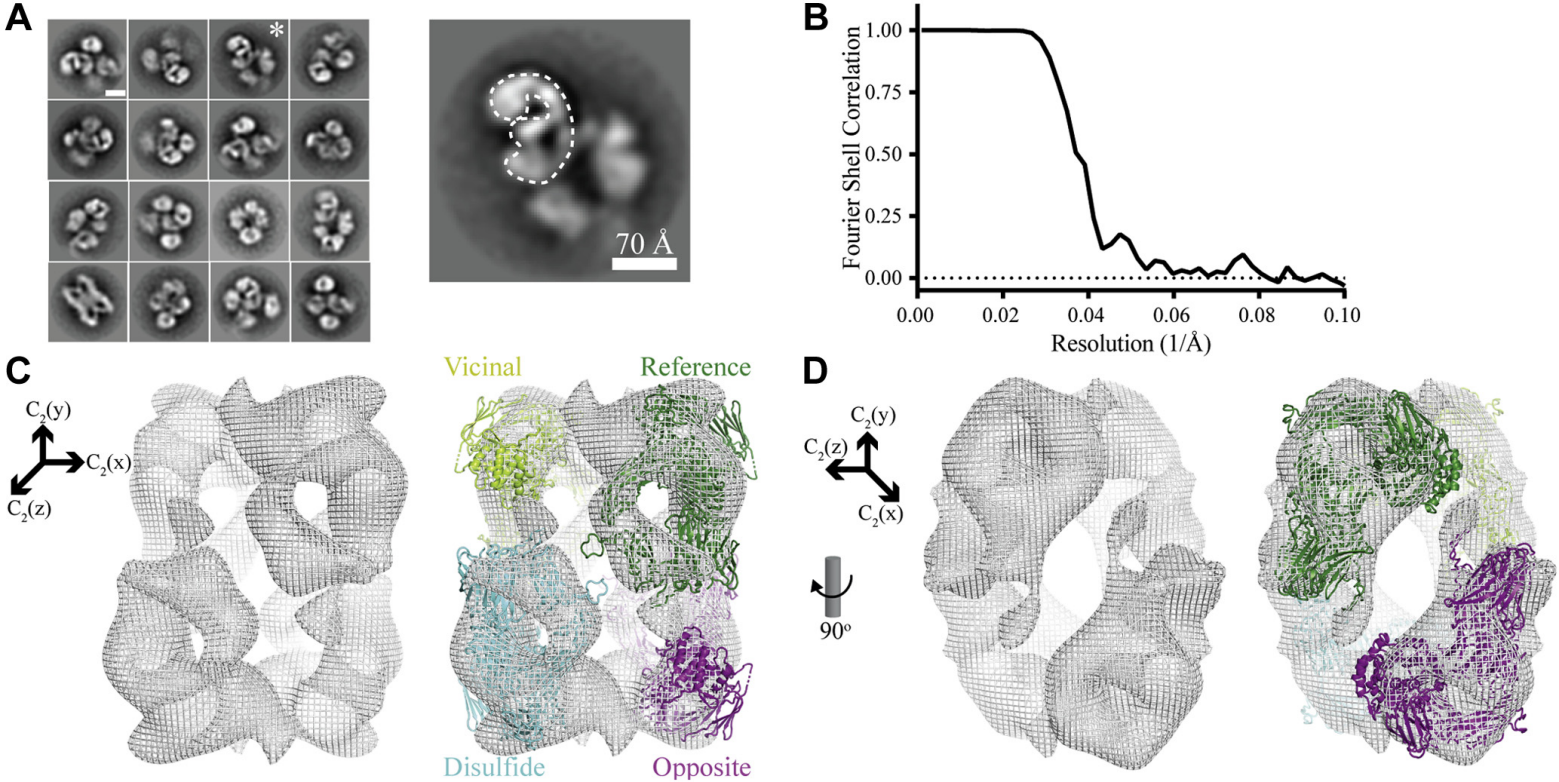
**Negative stain EM analysis of native A2M.***A*, selected 2D classes showing tetrameric native A2M in different orientations. A putative subunit is outlined in the magnified view of the 2D class marked by ∗ shown to the *right*. *B*, the corrected Fourier shell correlation (FSC) as a function of resolution (Å^−1^), calculated using Relion (35). The mask for FSC calculation was generated with lowpass filter set to 12 Å, a binarization threshold of 0.011, an extended binary map of ten pixels, and a soft edge of ten pixels. The masked, unmasked, and phase-randomized FSCs are shown together with the corrected FSC in supplemental Fig. S1*D*. *C* and *D*, different views of the 3D reconstruction (*left*) and with the A2M tetramer model superimposed and subunit assignment (*right*). The Cartesian coordinate systems indicate the direction of three C2 twofold rotation axes located in the A2M center of mass. One subunit is designated as the “reference” subunit (*green*) that participates in intersubunit disulfides with the subunit labeled “disulfide” (*cyan*), forming the disulfide-bridged dimer. The “opposite” subunit (*magenta*) interacts with the reference subunit through an interface formed by the LNK region of both subunits, although this interaction is not modeled here.

Negative stain EM was used to investigate the spatial arrangement of plasma-purified human A2M in its native conformation. 2D classes showing tetrameric A2M were manually selected (Fig. 1*A*) and had preferred orientations relative to the grid (supplemental Fig. S1*C*). 3D refinement with D2 symmetry gave a map with a calculated resolution of 24 Å (Fig. 1*B*, supplemental Fig. S1*D*).

**Fig. 2:**
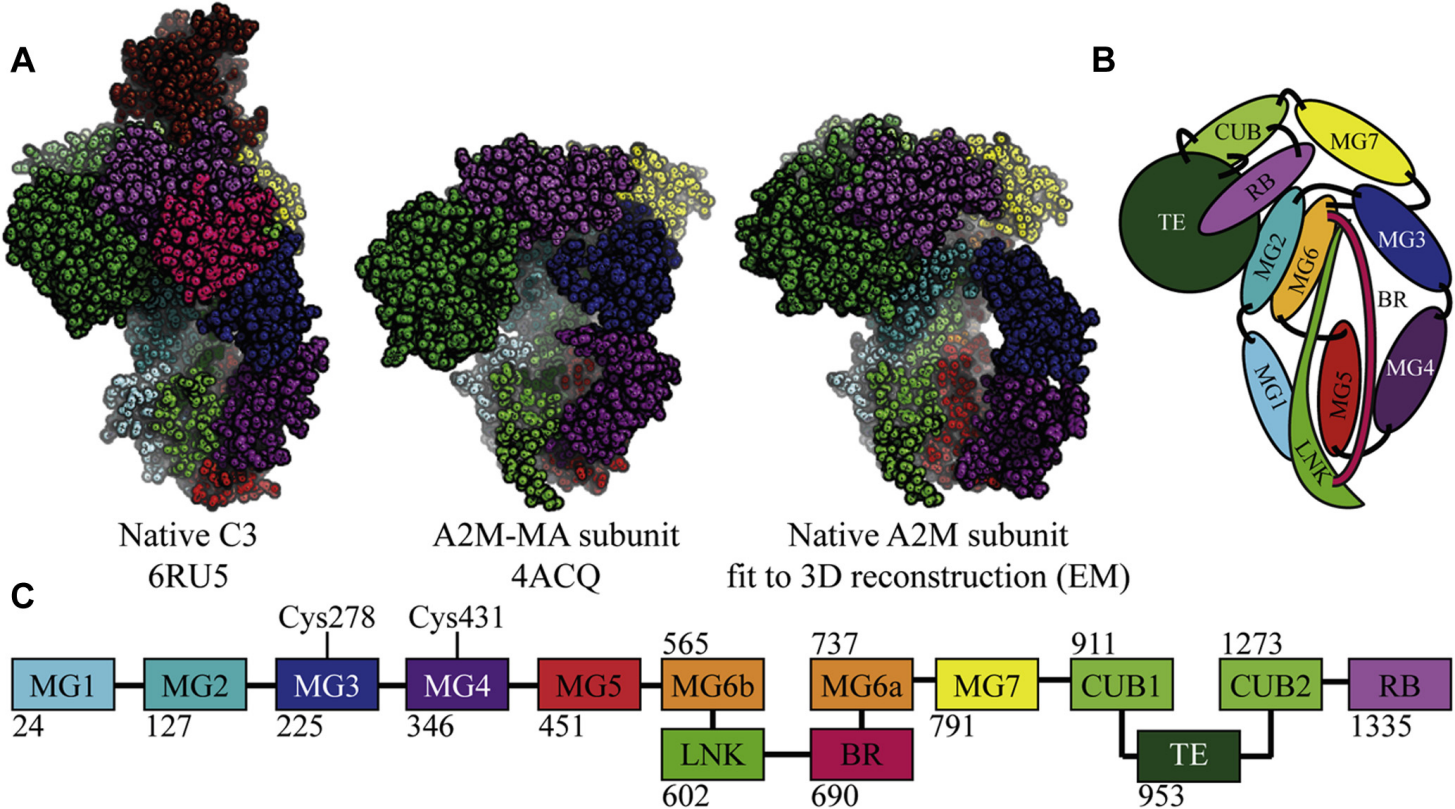
**Constructing an initial model for the native A2M subunit.***A*, the crystal structures of native C3 and a complete A2M-MA subunit are shown, as well as the model of the native A2M subunit created by alignment to C3 and fitting to the EM 3D reconstruction, as described in supplemental Fig. S1. *B*, a schematic illustration of the native A2M subunit model with labeled domains. *C*, schematic representation of the domains of A2M. Numbers indicate the starting residue of a domain, by the definitions used in this study. Cys278 and Cys431, which form two Cys278/Cys431 disulfides in each disulfide-bridged dimer, are indicated.

The crystal structures of C3 and C4 in their native and protease-cleaved conformations revealed a conformational change involving significant movements of the TE, CUB, and MG8 domains (11, 12, 13, 21, 22). For A2M, there is only a crystal structure of A2M-MA, a proxy of its protease-cleaved conformation (23). Therefore, in order to interpret the EM map and prepare a starting model for SAXS rigid-body refinement (see below), we constructed a model of the native A2M subunit by moving the domains in the crystal structure of A2M-MA to their equivalent positions in native C3 (Fig. 2). This native subunit model was fit to the EM map in Chimera using a correlation search, whereas the MG3 and MG4 domains were fitted manually to the 3D reconstruction as described in Supplemental Methods and shown in supplemental Fig. S1. The correlation between a map predicted by Chimera from this fitted model and the experimentally derived map was 0.59, and this relatively low correlation reflects several regions of the 3D reconstruction that are not accounted for by the model and vice versa (Fig. 1, *C* and *D*). The EM-based model of native A2M must therefore be considered tentative and is not necessarily unique due to the EM map’s low resolution. The model is used in this study as the basis for further analysis by SAXS and XL-MS. That said, the torus shape of the MG ring (MG domains 1–6) and the gap between the MG2, MG6, MG7, TE, and RB domains, which are characteristic structural elements of native αM proteins, are readily discernable in the density map despite the low resolution.

In the model of the native A2M tetramer obtained by fitting to the EM 3D reconstruction, the directions of the subunits alternate with respect to the major axis of the tetramer (Fig. 1, *C* and *D*). The TE and RB domains are located at the periphery of the tetramer. The MG rings of the disulfide-bridged dimers form two centrally located and slightly curved structures surrounding the central cavity in the equatorial plane of the molecule. The crystal structure of A2M-MA (23) revealed that any given reference subunit relates to each of the other three subunits in a distinct way: the reference subunit has a “disulfide-linked” subunit with which it forms a disulfide-bridged dimer and has a parallel “vicinal” and an antiparallel “opposite” subunit from the other disulfide-bridged dimer. We maintain this nomenclature for describing the subunits of native A2M, but the EM model shows distinct and much sparser interactions between subunits than in collapsed A2M, as elaborated by the XL-MS analysis described below.

### SAXS Analysis of A2M, A2M-MA, and A2M-T

**Fig. 3:**
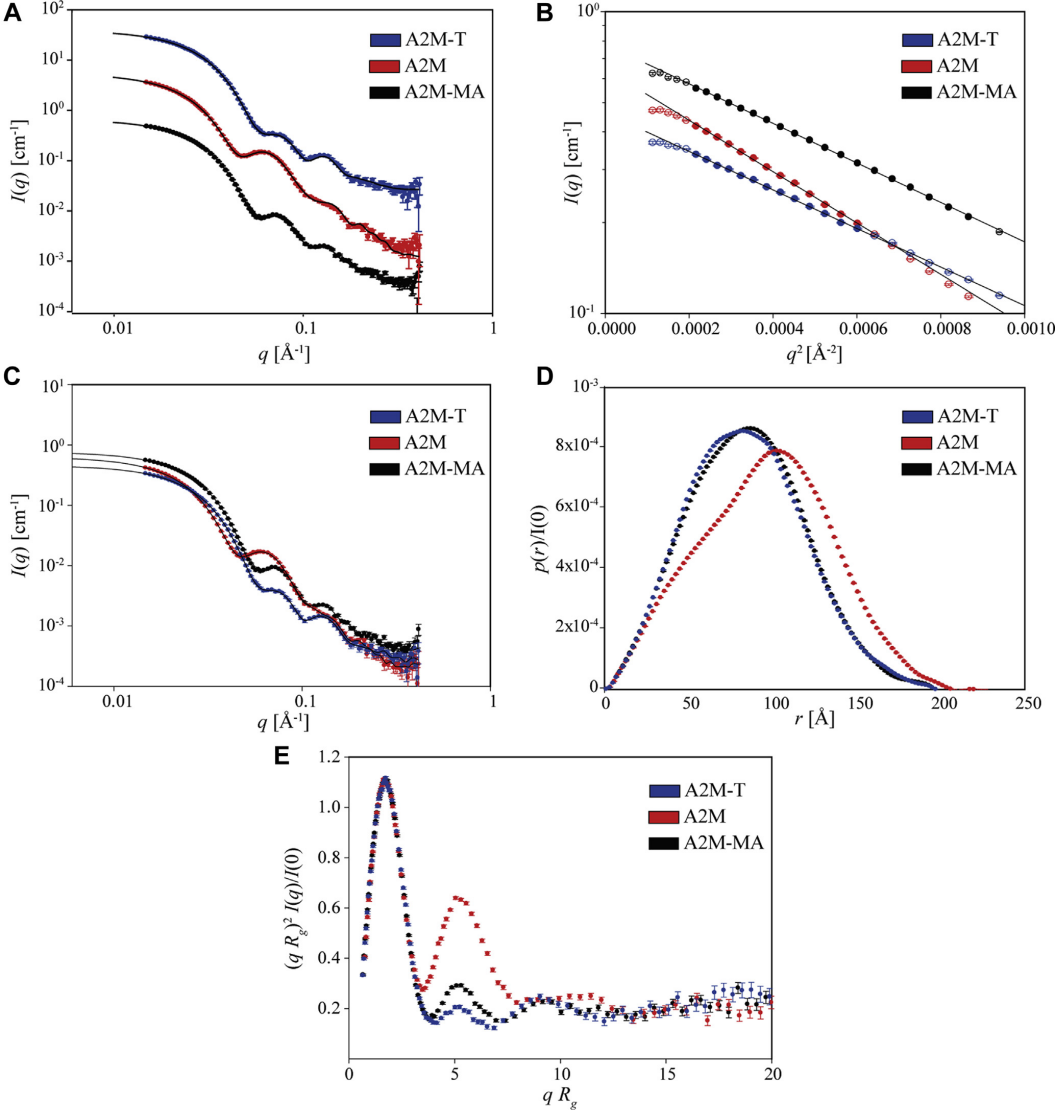
**Presentation and analysis of SAXS data from the glycosylated A2M samples.***A*, measured SAXS data and predicted SAXS curves from the most representative models obtained from rigid-body refinement (shown in Fig. 4). A2M and A2M-T data are offset 10-fold and 100-fold, respectively. *B*, Guinier plots and fits. Only data points with full symbols are included in the fits. *C*, SAXS data and indirect Fourier transformation fits. *D*, pair distribution functions from indirect Fourier transformation, normalized to I(0). *E*, dimensionless Kratky plot of the SAXS data.

In order to evaluate whether the EM model of native A2M was representative of its particle characteristics in solution, SAXS was measured for glycosylated A2M (Fig. 3*A*) and for A2M with glycans removed by deglycosidases (supplemental Fig. S2*A*), including methylamine- or trypsin-treated A2M for comparison. All data sets showed pronounced secondary oscillations, which demonstrated that the particles were monodisperse and globular. The SAXS data from the deglycosylated samples were very similar to the data collected for the glycosylated samples, suggesting that the structural influence of the glycosylations is modest (compare Fig. 3*A* and supplemental Fig. S2*A*). In addition, SAXS curves for A2M-MA and A2M-T were quite similar, while native A2M showed a more pronounced initial rise in its secondary oscillations, which is indicative of a greater degree of hollowness.

Guinier plots and fits for all data sets showed issues with normalization or background subtraction for points at the lowest values of the modulus of the scattering vector, *q* (Fig. 3*B*, supplemental Fig. S2*B*, supplemental Table S3). Subsequent data processing was therefore restricted to points with *q* >0.014 Å^−1^.

The fits from IFT and the corresponding distance distribution functions *p*(*r*) showed particle diameters of 20 to 21 nm for all samples. Native A2M had a triangular *p*(*r*) function, with the maximum shifted to a greater distance, further supporting its more expanded and hollow structure relative to A2M-MA and A2M-T (Fig. 3, *C* and *D*, supplemental Fig. S2, *C* and *D*). The molecular masses determined by Guinier fitting or IFT were within 2 to 6% of their theoretical values (supplemental Table S3). For both sets of A2M-MA and A2M-T samples, the Guinier radius of gyration is ~66 Å, whereas it is ~76 Å for native A2M, as determined by either Guinier fitting or *p*(*r*) functions. This contraction is consistent with the well-described conformational collapse of A2M when treated with proteases or methylamine (10, 42).

The dimensionless Kratky-plotted data of (*q R*_*g*_)^2^ *I*(*q*)/*I*(0) *versus qR*_*g*_, where *I*(*q*) is the SAXS intensity, all have a main maximum at $qR_{g}=\sqrt{3}$ with a height of 1.1 (Fig. 3*E*, supplemental Fig. S2*E*), as expected from relatively compact globular particles with a low content of flexible random coils (41). A secondary maximum is also observed for which the height increases with the hollowness of the structures, showing that A2M-T, A2M-MA, and native A2M are increasingly hollow, in that order.

The initial models for A2M-MA (based on the 4ACQ crystal structure) and native A2M (the model obtained by fitting to the EM 3D reconstruction) did not provide satisfactory fits to the SAXS data (weighted mean-square residuals, χ^2^, of 30.8 and 935, respectively). Fitting to the SAXS data from the deglycosylated samples was also unsatisfactory (χ^2^ = 14.2 and 1100 for A2M-MA and native A2M, respectively) and glycans were therefore not responsible for the poor fitting. Therefore, the models were optimized by rigid-body refinement.

### Rigid-Body Refinement of Models to SAXS Data

Restrained rigid-body refinement against the SAXS data was used to optimize the starting models for native A2M, A2M-MA, and A2M-T. As our rigid-body refinement is not deterministic, ten separate repetitions of rigid-body refinement were carried out for each data set. The ten models obtained for each sample showed little internal variation. For each sample, the most representative model with the lowest average RMS values compared with the other nine models was selected from the ten output models. The scattering curves calculated from these models fit well to the experimental data, with χ^2^ values of 1.3 to 3.7 (Fig. 3*A*, supplemental Fig. S2*A*). Protein concentrations calculated from the SAXS curves were within 5 to 14% of the concentrations determined from absorbance at 280 nm (supplemental Table S4).

**Fig. 4:**
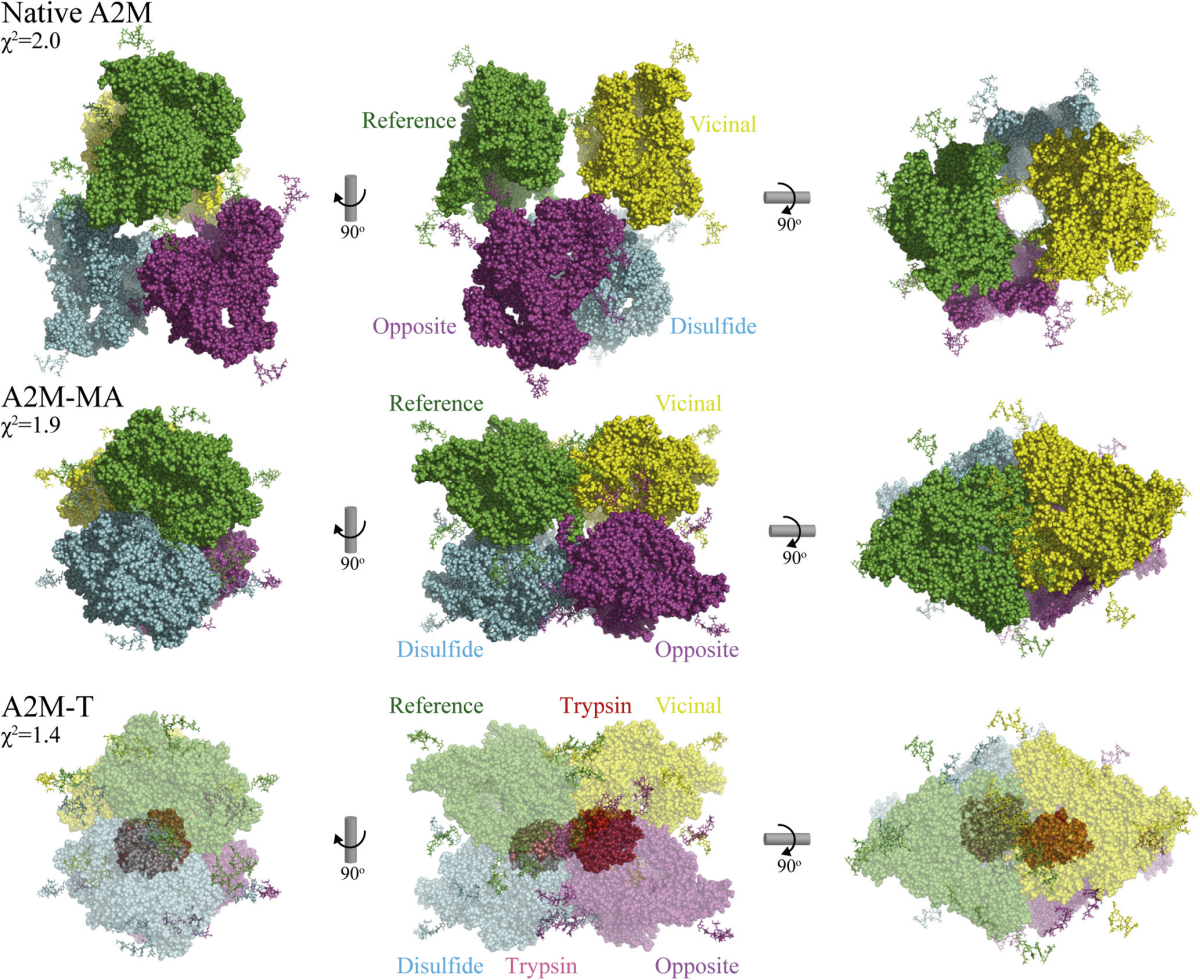
**SAXS-derived models of A2M.** Models of A2M were obtained by rigid-body refinement against the SAXS data from glycosylated A2M, A2M-MA, and A2M-T. The most representative model from ten nondeterministic refinement runs per conformation is shown. The A2M tetramers of each conformation, to the same scale, are shown from three angles with individually colored subunits. The χ^2^ of each model’s SAXS curve to the experimental SAXS data is given. Glycans are shown as *sticks*. A2M-T is shown with a partially transparent A2M to make the two trapped trypsin proteases visible. The reference subunit (*green*) is disulfide-bonded to one subunit (*cyan*), interacts through a symmetric LNK/LNK interface with an opposite subunit (*purple*), and is closely positioned to a vicinal subunit with which it likely forms a protease-inhibiting pair (*yellow*). In native A2M, the vicinal subunits are apart, while in A2M-MA and A2M-T, they have collapsed against each other, thereby closing off the A2M interior. The configuration of the disulfide-linked dimers similarly changes from an elongated crescent with minimal intersubunit contact to become more compact.

The most representative models obtained for A2M-MA and A2M-T were very similar to the initial model based on the A2M-MA crystal structure (Fig. 4, supplemental Fig. S3), as reflected by the low RMS values of 3.3 to 5.2 Å between the initial and refined models. The inter-Cα distances for the interdimer disulfides were 5.9 to 6.1 Å, tightly distributed around their target value of 6.0 Å from the initial model (supplemental Table S5). Similarly, the inter-Cα distances for contiguous residues in linkers connecting the rigid bodies used for fitting were 3.9 to 4.0 Å, compared with the target value of 3.8 Å derived from the starting model (supplemental Table S5). The low RMS values and inter-Cα distances show that the refined A2M-MA and A2M-T SAXS models maintain contiguity of the peptide backbone and are overall very similar to the A2M-MA crystal structure. This further supports the established structural similarity between A2M-MA and A2M that has been cleaved by small proteases.

For native A2M, the most representative rigid body-refined models had RMS values of ~14 Å to the EM-derived initial model (Fig. 4, supplemental Fig. S3), mostly due to changes in the positions and orientations of the MG7, CUB, and TE domains (supplemental Fig. S4). The inter-Cα distances for contiguous residues across the divisions of bodies were ~10 Å, with a target value of 3.8 Å. Both the higher RMS values and inter-Cα distances show that refinement introduced more deviations into the initial model of native A2M than in A2M-MA or A2M-T, which is expected considering the lower resolution of the data on which the EM-derived initial model of native A2M was based and the conservative assumption of a C3-like domain organization imposed during construction of this model. As the MG3 and MG4 domains were not remodeled from their structures in A2M-MA, the intersubunit disulfides could not be used as a restraint during refinement. Instead, seven different intersubunit restraints were introduced to maintain the relative subunit positions of the EM model. These restraints had target values of 37 to 108 Å and deviated by 1 to 3 Å after refinement (supplemental Table S6), showing that the refined models preserved the overall tetrameric organization identified by EM. These results show that the overall tetramer organization of native A2M identified by fitting to the EM reconstruction is well supported by the SAXS measurements. However, the SAXS rigid-body refinement introduced more extensive changes to the initial model of native A2M than for either collapsed A2M conformation. This indicates that the EM-derived initial model of native A2M is less accurate than the X-ray crystallography-derived initial models for A2M-MA and A2M-T.

### Analysis of Cross-linked A2M by PAGE

**Fig. 5:**
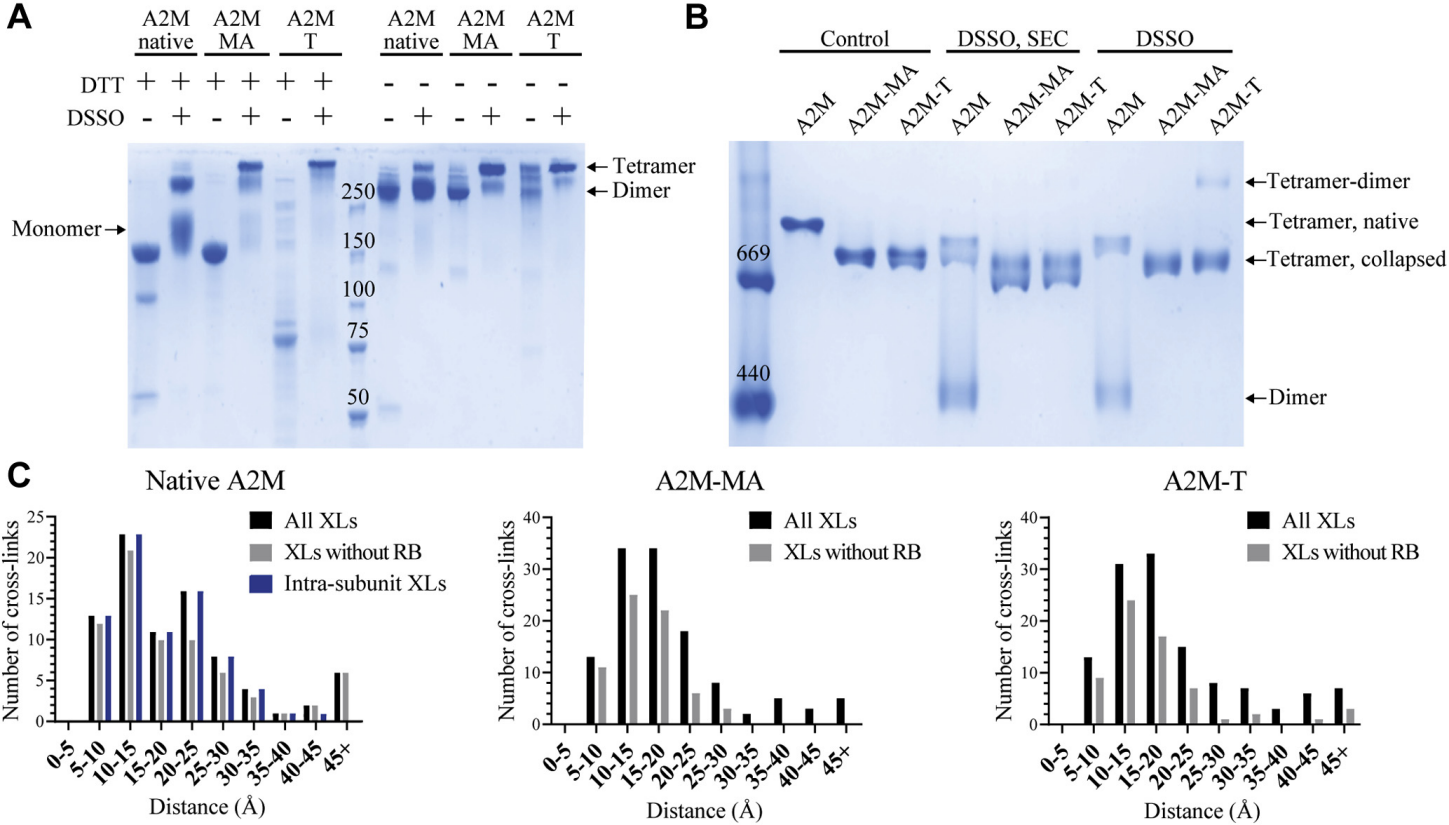
**Cross-linking of A2M with DSSO.***A*, DTT-reduced and nonreducing SDS-PAGE and *B*, native pore-limited PAGE of A2M in its three conformations, with and without DSSO cross-linking. In pore limit, the cross-linked A2M samples are shown before and after SEC purification. A2M-MA and A2M-T are cross-linked more between their subunits than native A2M. The cross-linked native A2M tetramer dissociates to its disulfide-bridged dimers on native PAGE but is a stable tetramer in solution (*i.e.*, SEC), as demonstrated in supplemental Fig. S5. A small amount of A2M-T is cross-linked to tetramer-dimers, which were removed by SEC. The SDS-PAGE migration of native A2M’s monomer, dimer, and tetramer bands, which were analyzed by XL-MS are indicated. *A* and *B*, the images shown are representative of multiple experiments. *C*, histograms showing the distribution of the inter-Cα distances for cross-links identified from DSSO-cross-linked A2M samples, measured using the SAXS-derived models. Interlysine DSSO cross-links have a maximum length of 26 Å, and longer distances therefore reflect a disparity between the model and the experimentally cross-linked protein. The majority of >26 Å distances in A2M-MA and A2M-T involve the RB domain, which is dynamic in these conformations and therefore imperfectly represented by a single position in their SAXS models. Intrasubunit cross-link lengths for native A2M show that intersubunit cross-links in the MG3 and MG4 domains account for all six of the >45 Å cross-links.

A third structural method, XL-MS, was used to unambiguously identify proximal lysine residues and evaluate whether these were consistent with our models. Cross-links were introduced in native A2M, A2M-MA, and A2M-T using disuccinimidyl sulfoxide (DSSO), an amine-reactive cross-linker with an MS-cleavable sulfoxide group (43). SDS-PAGE showed that native A2M was mostly cross-linked intrasubunit and between the subunits of disulfide-bridged dimers, while relatively little cross-linking was observed between subunits not linked by disulfide bridges (Fig. 5*A*). A2M-MA and A2M-T were more readily cross-linked across the entire tetramer (Fig. 5*A*). Native pore-limited PAGE showed dimerization of A2M-T tetramers and dissociation of native A2M tetramers into dimers after cross-linking (Fig. 5*B*). Therefore, size-exclusion chromatography (SEC) was performed on all cross-linked A2M samples to isolate A2M tetramers prior to further XL-MS studies. Cross-linked native A2M eluted as a tetramer during SEC, indicating that while cross-linking destabilized the native tetramer during native PAGE, it remained tetrameric in solution (supplemental Fig. S5, *A* and *B*).

### Identification of Intra- and Intersubunit Cross-links in Native A2M by In-gel Digest and XL-MS

To distinguish between intra- and intersubunit cross-links, in-gel digests were performed on the monomer band of cross-linked native A2M in reducing SDS-PAGE and on its dimer and tetramer bands in nonreducing SDS-PAGE (Fig. 5*A*). From these samples, 65 intrasubunit cross-links were identified from the monomer band and an additional ten unique cross-links were exclusively identified in the dimer band (Supplemental Spreadsheet 1). No unique cross-links were identified from the tetramer band. Label-free quantification was used to further ensure that the cross-links exclusively identified in the dimer band were not present in the monomer band. This identified six cross-links that were unique to the dimer and tetramer bands and therefore classified as intersubunit between disulfide-linked subunits, as well as one cross-link that is both intra- and intersubunit (supplemental Fig. S6, *A* and *B*). All identified intersubunit cross-linking of native A2M occurred between the MG3 and MG4 domains, *i.e.*, the known interface between disulfide-linked subunits.

### Cross-link Identification in Total Protein Digests of Native A2M, A2M-MA, and A2M-T

Total protein digests of native A2M, A2M-MA, and A2M-T were analyzed by XL-MS for a comparative study. Using this approach, 83 total intra-A2M cross-links were identified for native A2M, 125 for A2M-MA, and 117 for A2M-T. An additional 15 cross-links were identified between trypsin and A2M in the A2M-T complex. All cross-links are summarized in Supplemental Spreadsheet 1.

In order to evaluate whether XL-MS supported the SAXS-derived A2M models, cross-links were mapped onto the SAXS models in which they were identified and their lengths were assessed according to DSSO’s maximum inter-Cα distance of 26 Å (28). The shortest of each cross-link’s four possible positions was considered, except where the intra- or intersubunit nature of the cross-link in native A2M had been determined by in-gel digest. When considering all identified cross-links, this gave average inter-Cα cross-link lengths of 20.6 Å for native A2M, 19.6 Å for A2M-MA, and 21.2 Å for A2M-T; the distributions of cross-link lengths show that the XL-MS results were in general agreement with the SAXS models, considering the 26 Å length maximum (Fig. 5*C*). However, all of the intersubunit cross-links that were identified between disulfide-linked subunits in native A2M had lengths over 40 Å, indicating a conflict between the SAXS model and the XL-MS data. These cross-links involved the extended flexible loops in the MG3 and MG4 domains, which stretch across subunits to facilitate the intersubunit disulfides (MG3 residues 267–289, MG4 residues 423–443); as our data has insufficient resolution to allow remodeling of these loops, they were not changed from A2M-MA. Both the Cys278/Cys431 disulfides and these identified cross-links make it clear that the MG3 and MG4 loops must be restructured in native A2M. If only the intrasubunit cross-links in native A2M were considered, the average cross-link length in native A2M dropped from 20.6 to 17.2 Å and only two cross-links remained, which meaningfully exceeded 26 Å in length (Fig. 5*C*).

In A2M-MA and A2M-T, the RB domain was found to participate in a large number of cross-links exceeding 26 Å in length, indicating that the RB domain cannot be accurately represented by a single position in the two collapsed conformations. This mobility of the RB domain was also observed in the A2M-MA crystal structure (23). The agreement between the observed cross-links and the models of A2M-MA and A2M-T obtained by SAXS was therefore also assessed excluding cross-links involving the RB domain, which lowered their average cross-link lengths from 19.6 and 21.2 Å to 15.2 and 17.9 Å, respectively (Fig. 5*C*). In contrast, the lengths of RB domain cross-links in native A2M were generally below 26 Å (Fig. 5*C*), with an average length of 20.6 Å compared with their average length of 26.8 Å in A2M-MA. This supports a fixed position of the RB domain in native A2M that is consistent with a C3-like domain organization.

### Intrasubunit Cross-links in Native A2M Support a Native C3-like Domain Arrangement

**Fig. 6:**
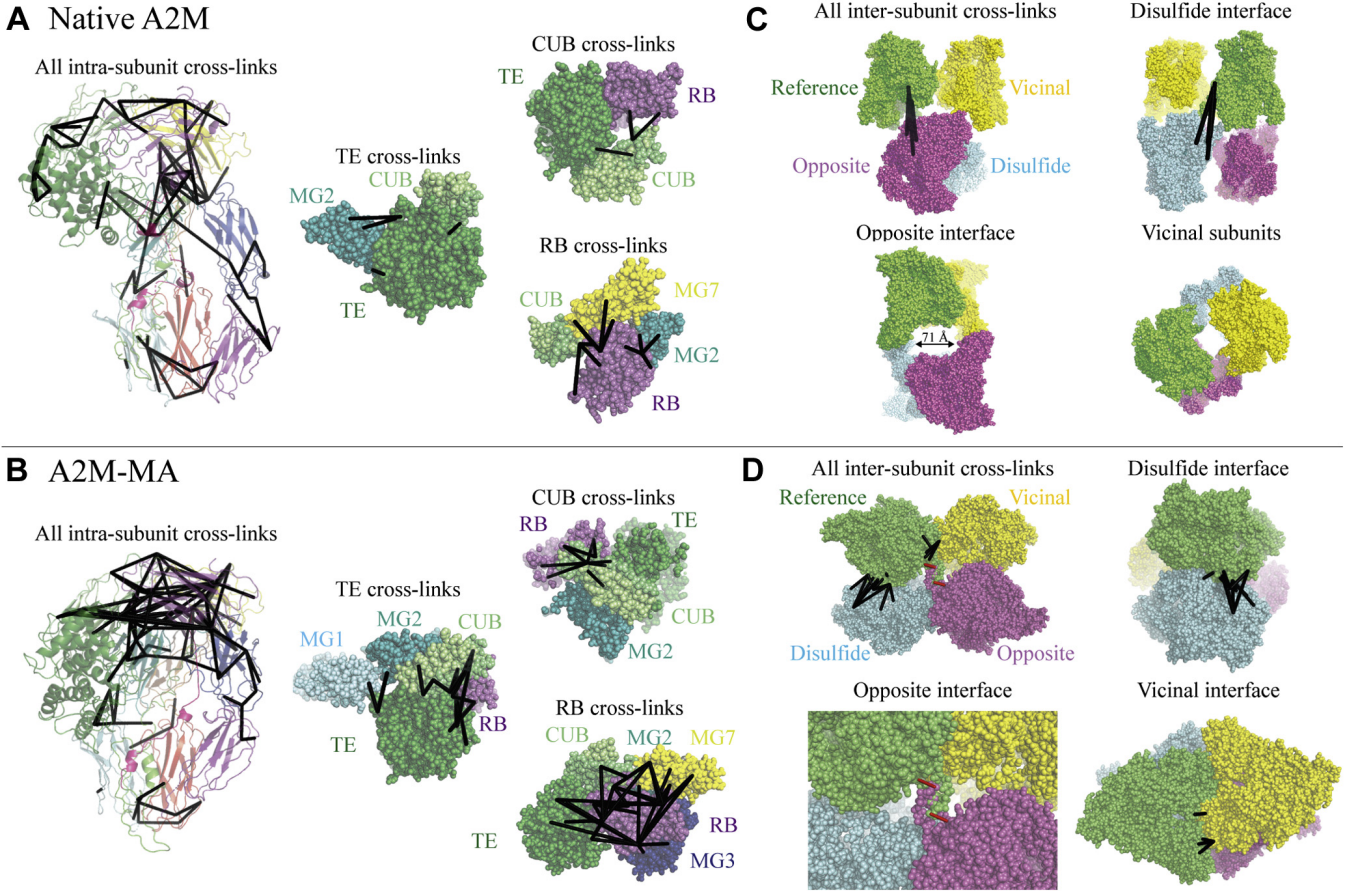
**Visualization of intra- and intersubunit cross-links identified for native A2M and A2M-MA.** Cross-links identified in native A2M and A2M-MA are represented in their respective models. Additional cross-link information can be found in Supplemental Spreadsheet 1. *A* and *B*, all intrasubunit cross-links identified in native A2M and A2M-MA are represented as black lines on a single subunit. *Insets* show only the domains and cross-links involving the TE, CUB, or RB domain, as labeled. *C* and *D*, all intersubunit cross-links identified in native A2M and A2M-MA are represented in their tetrameric models, colored by subunit. Panels focus on the three different intersubunit interfaces, as labeled, with DSSO cross-links shown as *black lines* and the disulfides introduced in the A2M^LNK/LNK^ mutant as *red lines*. Cross-links are shown originating from the reference subunit only. The only intersubunit cross-links identified in native A2M were between disulfide-linked; however, the A2M^LNK/LNK^ mutant demonstrates that the LNK regions of opposite subunits interact, spanning the ~70 Å distance between these subunits, as indicated. The lack of cross-links between vicinal subunits in native A2M despite the identification of three intervicinal cross-links in collapsed A2M supports the distant positions of these subunits in the native A2M models obtained by EM and SAXS.

Our model of native A2M assumes that it has a domain organization similar to native C3, but this cannot be demonstrated by low-resolution EM and SAXS data. Intrasubunit cross-links were therefore used to evaluate the validity of this assumption. The apical position of the TE domain relative to the MG ring in native A2M was supported by exclusive cross-links from the TE domain to the MG2 and CUB domains (Fig. 6*A*). While cross-links were not found directly between the TE and RB domains in native A2M despite their predicted adjacency, the position of the RB domain in a fixed position close to the TE domain was supported by cross-links from the RB domain to the MG2, MG3, MG7, and CUB domains (Fig. 6*A*). In native A2M, the CUB domain was cross-linked to the TE and RB domains, but not the MG ring, consistent with a peripheral location (Fig. 6*A*).

In the collapsed A2M-MA and A2M-T SAXS models, the dynamic TE, CUB, and RB domains are located at different positions than in native A2M. This was supported both by cross-links unique to the A2M-MA and A2M-T samples and cross-links that were only identified in the native A2M sample (Fig. 6*B*), validating the distinct subunit conformations used in our native A2M and collapsed A2M models.

### Intersubunit Cross-links Indicate Changes in A2M’s Tetrameric Configuration Upon Conformational Collapse

Intersubunit cross-links between disulfide-bridged subunits were identified in both native A2M and collapsed A2M. SAXS and EM indicated that the disulfide-bridged dimers are reconfigured during A2M’s conformational collapse, shifting the MG rings of the two subunits from a planar, antiparallel orientation in native A2M to an inclined and more parallel orientation in collapsed A2M. Distinct intersubunit cross-links in the vicinity of the MG3 and MG4 domains supported these predicted differences (Fig. 6, *C* and *D*). Interactions between the MG3 and MG4 domains involve flexible loops (MG3 residues 267–289, MG4 residues 423–443), which we did not model in native A2M in this study, but which are sufficiently long to accommodate the intersubunit Cys278/Cys431 disulfides and the identified intersubunit DSSO cross-links.

The crystal structure of A2M-MA identified a symmetric interaction between the LNK regions (residues 640–664) of opposite subunits, taking place across the disulfide dimers (23), and this is preserved in our A2M-MA and A2M-T SAXS models (Fig. 6*D*). The distance between opposite subunits and their orientation to each other would allow this interaction to take place in native A2M according to our model (Fig. 6*C*), although we have not remodeled the LNK regions in native A2M. In fact, there are no modeled interacting surfaces between the disulfide dimers in our native A2M models, which could indicate that interacting LNK regions are solely responsible for holding the A2M tetramer together. However, there are no lysine residues in the interacting part of the LNK region. This intersubunit interaction was therefore not demonstrated by cross-links identified from any conformation of A2M, even A2M-MA in which it has been shown to take place (Fig. 6, *C* and *D*).

The A2M-MA crystal structure shows a third intersubunit interface between vicinal subunits with large interacting surfaces involving the LNK, TE, and MG1 domains; cross-links corroborating this interface were identified in A2M-MA and A2M-T, but not native A2M, in agreement with our EM and SAXS models of native A2M where these subunits do not directly interact (Fig. 6, *C* and *D*).

Neither SAXS nor XL-MS distinguished the structures of A2M-MA and A2M-T. The A2M-T SAXS model suggests trapping of the trypsin proteases within the prey chambers (supplemental Fig. S7*A*) that were previously identified in A2M-MA (23). However, these trypsin positions represent an average of many possibilities, as proteases are heterogeneously oriented when trapped by A2M (44). Accordingly, the cross-links identified between A2M and trypsin in A2M-T were not compatible with a single trypsin location and orientation. Instead, they supported a possibility space for trapped trypsin centered on its placement identified by SAXS (supplemental Fig. S7*B*). Cross-links involving the neo-N-terminus of the cleaved bait region suggested that the N-terminus of the bait region migrates outside the tetramer after cleavage, similarly to the relocation of the α’ chain’s N-terminus observed during the conformational transition from complement C3/C4 to C3b/C4b (22).

### Investigating the Bait Region Position and LNK Region Interface Using A2M Mutants

XL-MS was able to validate the C3-like domain organization of a native A2M subunit and supported the intersubunit relationships in our native A2M model. However, there was no coverage of the bait region or the LNK region in XL-MS analysis of plasma-purified A2M due to their lack of lysine residues. Determining the bait region location is critical to understanding the inhibitory mechanism of A2M, and symmetrical interactions between the LNK regions of opposite subunits could be responsible for formation of the A2M tetramer. To further investigate these regions, we recombinantly expressed two A2M mutants. In the first mutant, A2M^3K^ (R704K R715K R719K), the three bait region arginine residues are replaced with lysines, allowing the position of the bait region to be investigated using XL-MS with DSSO. In the second mutant, A2M^LNK/LNK^ (T654C T661C), a disulfide was introduced across the two opposite subunits in the symmetrically interacting LNK regions in order to demonstrate their proximity.

Reducing SDS-PAGE showed that both A2M^3K^ and A2M^LNK/LNK^ underwent thiol ester-dependent heat fragmentation to 120 kDa and 60 kDa fragments, were preferentially cleaved in the bait region by trypsin, and covalently conjugated trypsin (supplemental Fig. S8, *A* and *C*). A2M^3K^, but not wild-type A2M, was cleaved in its bait region and conjugated to LysC, a lysine-specific protease (supplemental Fig. S8*A*). Native pore-limited PAGE showed that both mutants initially had the electrophoretic mobility of native A2M and collapsed upon MA treatment or bait region cleavage (supplemental Fig. S8, *B* and *D*). These results show that A2M^3K^ and A2M^LNK/LNK^ retain the structure and functionality of wild-type A2M.

**Fig. 7:**
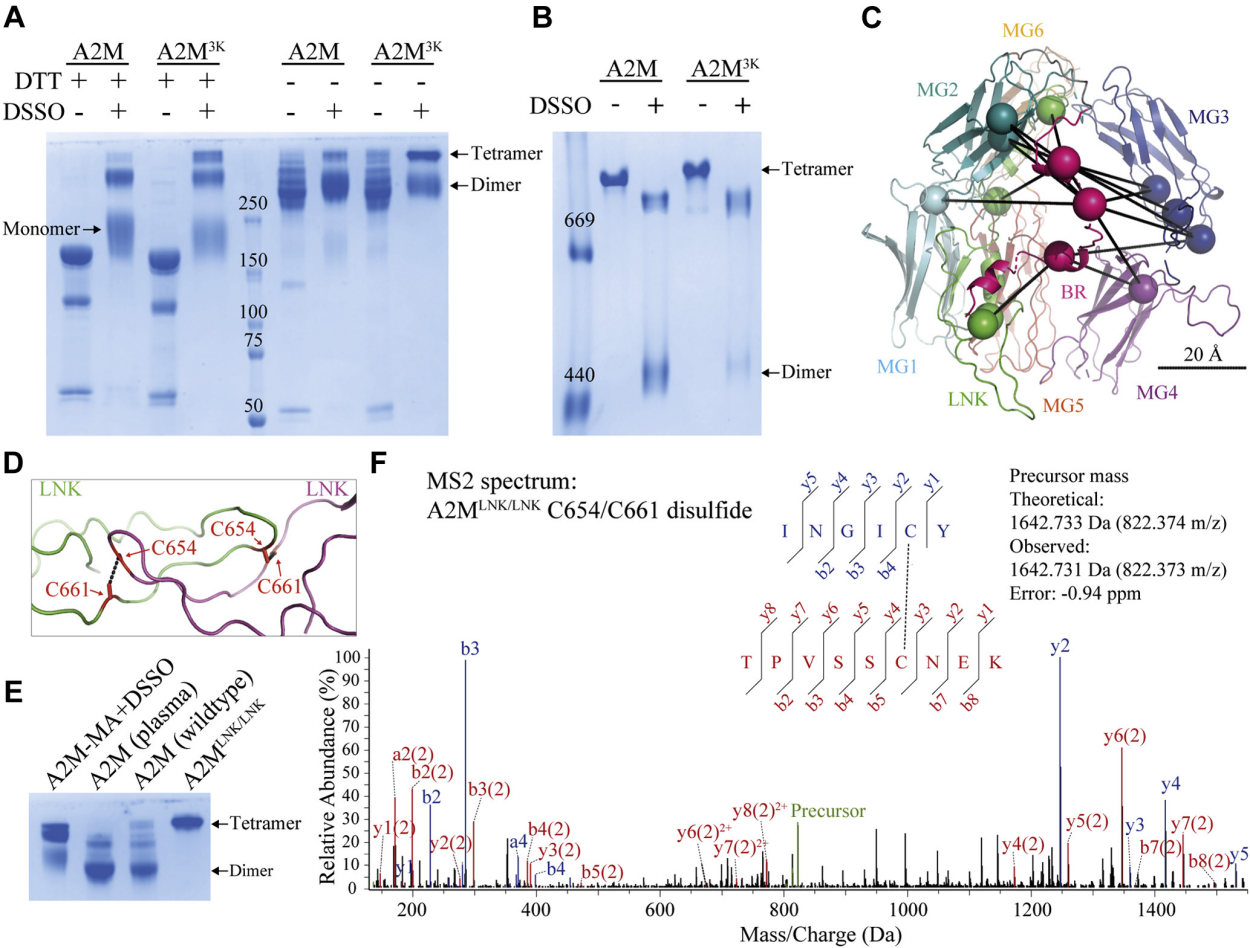
**Validation of the native A2M model with the A2M**^**3K**^**and A2M**^**LNK/LNK**^**mutants.***A*, reducing and nonreducing SDS-PAGE and *B*, pore-limited native PAGE of A2M and A2M^3K^ with and without DSSO cross-linking. A2M^3K^ is more readily cross-linked across its disulfide dimers. *C*, cross-links identified from the A2M^3K^ monomer band are on only the MG ring of the native A2M EM model. Residues participating in cross-links are shown as *spheres*. The bait region is modeled in a position spanning the MG ring and oriented toward the interior of the A2M tetramer in order to accommodate these cross-links. A 20 Å scale bar at the same depth as the bait region is shown. Additional cross-link information is given in supplementary Spreadsheet 1. *D*, the symmetric interactions between LNK regions of opposite subunits, as seen in the 4ACQ structure of A2M-MA. The novel disulfides introduced in A2M^LNK/LNK^ are indicated. *E*, nonreducing SDS-PAGE of DSSO-cross-linked A2M-MA (included to define the migration of dimer and tetramer A2M bands), plasma A2M, recombinant wild-type A2M, and recombinant A2M^LNK/LNK^. A2M^LNK/LNK^ shows a sharp tetramer band, indicating that at least one LNK/LNK-bridging disulfide is formed per tetramer. *F*, a MS2 spectrum identified a disulfide-linked peptide covering the cysteine residues introduced by mutation from A2M^LNK/LNK^ digested with both trypsin and chymotrypsin. Fragment ions from the INGICY peptide are blue and fragments from TPVSSCNEK are *red*. Other peptides covering this disulfide, obtained by digestion with chymotrypsin alone or pepsin, are shown in supplemental Fig. S9.

A2M^3K^ was cross-linked by DSSO similarly to native A2M, although a greater extent of interdisulfide dimer cross-linking is apparent from the increased tetramer band intensity (Fig. 7*A*). This indicates that the bait region can be positioned close to other subunits. As with native A2M, cross-linked A2M^3K^ partially dissociated during native electrophoresis (Fig. 7*B*), but eluted as a tetramer in SEC. An in-gel digest of cross-linked A2M^3K^’s monomer gel band with subsequent MS-based cross-link identification was performed, as described for native plasma-purified A2M. A total of 19 intrasubunit bait region cross-links were identified (Supplementary Spreadsheet 1), showing cross-linking of the bait region to the inward-facing side of a native A2M subunit’s MG ring, oriented toward the tetramer’s interior (Fig. 7*C*).

In A2M^LNK/LNK^, a disulfide was introduced across the two opposite subunits by the T654C and T661C mutations, chosen due to their inter-β-carbon distance of 3.8 Å in the A2M-MA crystal structure (Fig. 7*D*). In nonreducing SDS-PAGE, A2M^LNK/LNK^ migrated exclusively as a tetramer, in contrast to dimer-migrating plasma-purified A2M and recombinant wild-type A2M, indicating that new disulfides were introduced, which linked the existing disulfide-bridged dimers (Fig. 7*E*). Low-pH digests of A2M^LNK/LNK^ were analyzed by LC-MS/MS and disulfide-bonded peptides confirming both disulfide formation between Cys654 and Cys661 and the intersubunit nature of these disulfides were identified in three separate protease digests, giving coverage of the disulfide with multiple peptides (Fig. 7*F*, supplemental Fig. S9). The introduction of an intersubunit disulfide across LNK regions in native A2M without changing its structure or functionality strongly supports the existence of this interface in native A2M.

### A Model of Protease Trapping by A2M

To present our SAXS-derived models and visualize A2M’s conformational change, we have interpolated the native and collapsed conformations (supplemental Movie S1). All intersubunit relationships (*e.g.*, disulfide-linked, vicinal, and opposite) are preserved during the conformational change, as is required by the persistent Cys278/Cys431 disulfides and LNK/LNK interfaces (although these are not modeled).

**Fig. 8:**
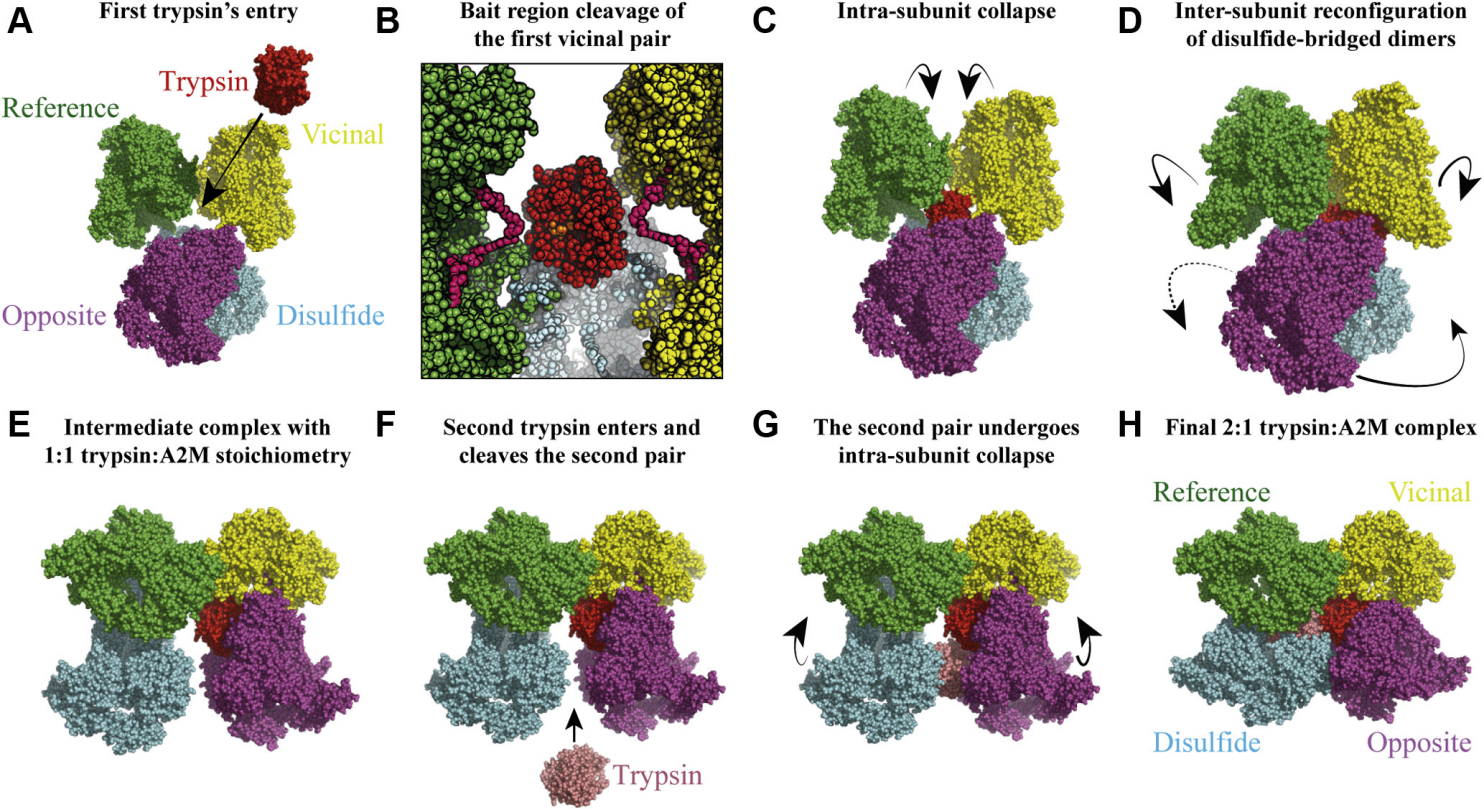
**A putative model for protease trapping by A2M.** The trapping of two trypsin proteases by A2M was modeled using the SAXS-derived models of native A2M and A2M-T. This trapping model assumes that the subunits that are vicinal to each other form a protease-inhibiting dimer. *A*, the first trypsin (*red*) enters the interior of native A2M and *B*, cleaves the bait regions (modeled and colored *pink* in this panel only) of the first vicinal pair (*green* and *yellow*); the bait regions occupy the central space of the pair and can plausibly be cleaved in rapid succession. *C*, bait region cleavage triggers the intrasubunit conformational change (*i.e.*, TE domain migration) in the vicinal pair. *D*, the collapsed vicinal subunits can now interact through their TE domains, driving a reconfiguration of the disulfide-bridged dimers, which also moves the second vicinal pair (*cyan* and *purple*). *E*, the metastable intermediate complex between a single trypsin and A2M. The first vicinal pair is fully collapsed, while the subunits of the second vicinal pair have a native intrasubunit conformation but are placed in their A2M-T positions. *F*, the second trypsin (*salmon*) enters A2M and cleaves the bait regions of the second vicinal pair. *G*, the second vicinal pair undergoes the intrasubunit conformational change, giving (*H*) the final complex between fully collapsed A2M and two trypsin proteases. This protease trapping model is animated in supplemental Movie S2.

A2M’s physiologically relevant conformational change, where it sequentially traps up to two proteases, is a two-step process with an intermediate 1:1 A2M:protease complex. We present a putative model for protease trapping by A2M based on our structural studies of native A2M and A2M-T (Fig. 8, supplemental Movie S2). Notably, this model of inhibition describes the context in which a protease encounters A2M’s bait region and initiates trapping by A2M and how half of the A2M tetramer might collapse while maintaining the tetramer. There are several underlying assumptions to this model, which should be noted. The model assumes that the vicinal subunit pairs comprise the minimal protease-inhibiting units. Furthermore, it assumes that bait region cleavage in the first-cleaved vicinal pair initiates a primarily intrasubunit conformational change in this pair. This then drives intersubunit rearrangements in the tetramer, so that the disulfide-bridged dimers have assumed their collapsed configuration in the intermediate 1:1 complex. These assumptions are consistent with the extant characterization of A2M, but may yet be challenged through structural studies of stabilized 1:1 complexes such as the A2M:plasmin complex (45, 46).

## Discussion

In this study, we combined negative stain EM, SAXS, and XL-MS to investigate the structure of native A2M and to compare to its methylamine- or trypsin-treated conformation. We have produced a plausible model of native A2M that is well supported by our complementary data. However, the low resolution of the three techniques must be kept in mind when interpreting our model.

One potential limitation in our approach is the assumption of D2 symmetry to avoid overfitting of low-resolution data. It is possible that the native A2M tetramer is dynamic and rarely or never adopts a state of perfect D2 symmetry. Another issue is the lack of information regarding the exact domain arrangement in native A2M; the C3-like configuration used in our model will not be entirely accurate. Furthermore, the domain structures were adopted from the A2M-MA crystal structure and may be different in native A2M; this is certainly the case for the MG3 and MG4 regions containing the intersubunit Cys278/Cys431 disulfides and for the LNK regions, all of which are extended loop regions in A2M-MA. Further validation and the elucidation of atomic details within this unique protease inhibitor will require high-resolution methods such as a single-particle cryoEM-based 3D reconstruction or the formation of a well-diffracting crystal of native A2M.

Native A2M shows a higher degree of susceptibility than collapsed A2M toward dissociation into its disulfide-bridged dimers, by either limited reduction or partial denaturation (10). Our model accounts for this difference, as it shows that the disulfide dimers appear to be held together mostly by the two symmetrical LNK/LNK interfaces in native A2M. In contrast, additional interactions occur across the vicinal MG1/TED interfaces in collapsed A2M. The sparsity of interactions in native A2M explains the relative ease by which it can be dissociated by reduction or denaturation *in vitro* and by oxidation occurring under physiological conditions such as inflammation (47), which is implicated in its chaperone-like activity (48) and may facilitate the delivery of nonproteolytic pathogenic proteins to antigen-presenting cells expressing LRP1 (49).

A2M’s interaction with the scavenging receptor LRP1 is dependent on its conformation. Native A2M does not interact with LRP1, whereas both A2M-MA and A2M-protease complexes do. A2M-protease complexes are therefore rapidly cleared from circulation by LRP1-expressing hepatocytes (50) and are opsonized by LRP1-expressing antigen-presenting cells (51). Similarly, collapsed A2M can interact with Grp78 on the surface of macrophages (52). A2M’s interactions with LRP1 and Grp78 are dependent on Lys1393 and Lys1397, respectively (53, 54), which are located on an α-helix in A2M’s RB domain (supplemental Fig. S10*A*). Our native A2M model indicates that the RB domain itself is positioned at A2M’s exterior toward the end of each subunit. However, due to the fixed position and orientation of the RB domain, the receptor-binding α-helix faces the interior of the tetramer and is inaccessible (supplemental Fig. S10*B*). A2M’s conformational collapse liberates the RB domain from its defined position, making the receptor-binding α-helix accessible from outside the tetramer.

The bait region of A2M is fundamental to A2M’s mechanism of inhibition, as a protease’s ability to cleave the bait region is the only known determinant to whether it is inhibited by A2M. The bait region is unstructured and poorly conserved across species (55). Accordingly, it appears disordered in the crystal structure of A2M-MA (23) and in structures of monomeric bacterial αMs (17, 56). Using A2M^3K^, the bait region position was determined to be proximal to the inward-facing surface of the A2M subunit. Furthermore, increased cross-linking of A2M^3K^ across the entire tetramer indicates that the bait region is close to other non-disulfide-linked subunits, supporting previously identified interactions between the bait regions of different subunits (57). These results experimentally validate the placement of the bait regions in our native A2M model, in the interior cavity of the A2M tetramer.

Inhibition of most proteases by A2M is a two-step process where A2M is cleaved by and subsequently traps two proteases (58), implying that two A2M subunits comprise the inhibitory unit. The functional division of A2M has not been conclusively identified; dissociation of A2M into dimers by limited reduction or denaturation yields two distinct types of “half-molecules,” but both retain some inhibitory capacity that is associated with tetramer reassembly (59). In our model of native A2M, the vicinal subunit pairs are aligned parallel to each other with adjacent bait regions and TEs and could plausibly be cleaved successively by the same protease. Furthermore, these subunit pairs cooperatively block each entrance to the hollow interior in collapsed A2M and their TEs are close enough to conjugate a single protease simultaneously (23). Interestingly, this vicinal subunit pair does not appear to directly interact in native A2M, and the A2M half-molecule produced by limited reduction is likely to be the LNK/LNK-interacting “opposite” subunit pair instead. This would explain the difficulty in identifying A2M’s inhibitory unit among its dissociated half-molecules.

In conclusion, we have constructed a model of native A2M based on structural information obtained using negative stain EM, SAXS, and XL-MS. This model structurally accounts for the unique trapping mechanism by which A2M inhibits proteases. Proteases must physically enter A2M to cleave a bait region, which triggers changes in both intrasubunit domain conformation and the configuration of the disulfide dimers to seal off a hollow interior in which the protease is trapped. This explains the independence of A2M’s protease trapping from covalent conjugation through the thiol ester (18), in contrast to monomeric αM protease inhibitors such as *E. coli* ECAM, which lack this enclosed interior and require covalent binding to inhibit proteases (17). A2M’s structural collapse brings the TE domains of vicinal subunits toward each other, thereby sealing the entrance to the interior space, which is occupied by bait regions. Our native A2M model supports a functional division of A2M as two vicinal, noncovalent subunit pairs that each independently trap a single protease, as illustrated in supplemental Movie S2. Other tetrameric inhibitors, including A2M from different species and related proteins such as ovostatin, share its inhibitory mechanism, and the model for inhibition described here is likely applicable to other tetrameric αM protease inhibitors.

## Data Availability

Mass spectrometry raw data and Proteome Discoverer result files (which contain all cross-linked peptide spectra) have been deposited to the ProteomeXchange Consortium *via* the PRIDE (60) partner repository with the identifiers PXD019101 (A2M, A2M-MA, and A2M-T), PXD019048 (A2M^3K^), and PXD019081 (A2M^LNK/LNK^). The SAXS data and models have been deposited in the SASBDB database (61) with the identifiers SASDJK3 (A2M), SASDJL3 (A2M-MA), SASDJM3 (A2M-T), SASDJN3 (A2M, deglycosylated), SASDJP3 (A2M-MA, deglycosylated), and SASDJQ3 (A2M-T, deglycosylated).

## Supplemental data

This article contains supplemental data (62, 63, 64, 65, 66, 67, 68, 69, 70).

## Conflict of interest

The authors declare that they have no conflicts of interest with the contents of this article.

## Supplemental Data

Supplemental Movie S2

Supplementary Spreadsheet 1
