# Supplementary material for: Structural Investigations of Human A2M Identify a Hollow Native Conformation That Underlies Its Distinctive Protease-Trapping Mechanism
Source: Mol Cell Proteomics. 2021 May 6;20:100090. doi: 10.1016/j.mcpro.2021.100090 (PMC8167298; doi:10.1016/j.mcpro.2021.100090)
Supplement: Supplementary Methods, Figures S1–S10 and Tables S1–S6 [file mmc4.docx]

**Supporting information**

**Structural investigations of human A2M identify a hollow native conformation that underlies its distinctive protease-trapping mechanism.**

Seandean Lykke Harwood^1,2^, Jeppe Lyngsø^3,4^, Alessandra Zarantonello^1^, Katarzyna Kjøge^1^, Peter Kresten Nielsen^2^, Gregers Rom Andersen^1^, Jan Skov Pedersen^3,4*^, Jan J. Enghild^1,3*^

^1^ Department of Molecular Biology and Genetics, Aarhus University, Aarhus 8000, Denmark,

^2^ Global Research Technologies, Novo Nordisk A/S, Novo Nordisk Park, Måløv 2760, Denmark.

^3^ Interdisciplinary Nanoscience Center, Aarhus University, Aarhus 8000, Denmark

^4^ Department of Chemistry, Aarhus University, Aarhus 8000, Denmark

**Supporting information in this document**

**Supplementary methods** S-2

[TABLE S1. **SAXS measurements and modeling – general information** S-4](#_Toc40878717)

[TABLE S2. **Rigid-body definitions used in model refinement against SAXS data** S-5](#_Toc40878717)

[TABLE S3: **Guinier fits and IFT results for the forward scattering, and Guinier radii** S-6](#_Toc40878723)

[TABLE S4. **Concentrations of samples determined from absorbance and SAXS modelling**. S-6](#_Toc40878721)

[TABLE S5. **Values of the distance restraints in the rigid-body refinement to SAXS data** S-6](#_Toc40878727)

[TABLE S6. **Additional restrains used for rigid-body refinement of native A2M SAXS models** S-7](#_Toc40878727)

[FIGURE S1. **Fitting the native A2M subunit model into the EM 3D reconstruction** S-8](#_Toc40878718)

[FIGURE S2. **Presentation and analysis of SAXS data from the deglycosylated A2M samples** S-9](#_Toc40878718)

[FIGURE S3. **SAXS-derived models of deglycosylated A2M** S-10](#_Toc40878720)

[FIGURE S4. **SAXS-derived models of A2M subunits** S-11](#_Toc40878720)

[FIGURE S5. **Size exclusion chromatography of DSSO-cross-linked A2M** S-12](#_Toc40878726)

[FIGURE S6. **Label-free quantification of A2M cross-links** S-13](#_Toc40878728)

[FIGURE S7. **Cross-links between A2M and trypsin** S-14](#_Toc40878728)

[FIGURE S8. **Basic functionality of the A2M^3K^ and A2M^LNK/LNK^ mutants.** S-15](#_Toc40878728)

[FIGURE S9. **Additional MS2 spectra of the novel A2M^LNK/LNK^ disulfide** S-16](#_Toc40878729)

[FIGURE S10. **Position of the receptor-binding site in native A2M** S-17](#_Toc40878729)

**Other supporting information:**

**Supplementary Spreadsheet 1 -** Contains information on all cross-links identified by XL-MS.

**Supplementary Movie 1 -** Visualizes native and collapsed A2M from 3 different angles.

**Supplementary Movie 2 –** Animates a putative model for the trapping of two small proteases by A2M.

**MS data –** Data from the A2M, A2M^3K^, and A2M^LNK/LNK^ samples have been deposited to the ProteomeXchange Consortium (60) under the identifiers of PXD019101, PXD019048, and PXD019081.

**SAXS data** – Models and measurements have been deposited to SASBDB (61) under the identifiers of SASDJK3 (native A2M, glycosylated), SASDJL3 (A2M-MA, glycosylated), SASDJM3 (A2M-T, glycosylated), SASDJN3 (native A2M, deglycosylated), SASDJP3 (A2M-MA, deglycosylated), and SASDJQ3 (A2M-T, deglycosylated).

**Supplementary methods**

### *Gene and plasmid preparation*

The plasmid encoding recombinant A2M was prepared by synthesizing a wildtype A2M gene based on the mRNA sequence M11311 (62) and inserting it into the pCDNA3.1(+) plasmid using *Nhe*I/*Xba*I restriction sites. Silent mutations were used to introduce *Hind*III and *EcoR*I sites flanking the bait region-encoding sequence, allowing it to be replaced with another synthesized sequence encoding a modified bait region with the Arg704Lys, Arg715Lys, and Arg719Lys mutations, giving the A2M^3K^ mutant. The Thr654Cys and Thr661Cys mutations were introduced by site-directed mutagenesis into the wildtype A2M plasmid to create the A2M^LNK/LNK^ mutant. All gene synthesis, cloning, and mutagenesis was performed by Genscript.

### *A2M model construction for SAXS rigid-body refinement*

The starting model for A2M-MA and A2M-T, which included the RB domain in all four subunits, was constructed by superimposing the single complete subunit present in RCSB entry 4ACQ (23) on the three other subunits. The missing regions in the bait region were built manually in PyMOL (63) and Coot (64) in a single subunit and transferred by superposition to the other three subunits. Modeled regions were adjusted in the monomer until no significant overlaps were present in the resulting tetramer. The tetramer model of A2M-MA was minimized with phenix.geometry_minimization (65). The starting model for native A2M was constructed by superposition of the structure of native complement C3 in RCSB entry 6RU5 (66) onto the MG1-6 and LNK domains of the single complete A2M-MA subunit from RCSB entry 4ACQ (23). The MG2, MG6, MG7, CUB, TE, and RB domains from A2M-MA were then superpositioned onto the equivalent domains in the native C3 molecule relative to MG domains 1-6 in A2M-MA to generate a model of a native A2M subunit with a C3-like domain configuration. Next, the resulting model of the native A2M monomer was fit as one rigid body to the 3D reconstruction from negative stain EM in Chimera (69). The MG3 and the MG4 domains were then fit to the density map manually in PyMOL, taking into account the connectivity with the MG2 and MG5 domains and the inter-subunit disulfide bridges formed by Cys278-Cys431 between the subunits of the disulfide-bridged A2M dimer. Finally, the native A2M tetramer was generated by application of D2 symmetry. Note that residues 626-662 of the LNK region and residues 690-728 of the bait region were not included in the native A2M model. Glycosylations were added using the program GLYCOSYLATION from the ATSAS package (67), using the previously determined glycosylation sites (26) and glycans of appropriate mass.

### *SAXS rigid-body refinement*

Rigid-body refinement was performed using home-written software (68,69). The program calculates the scattering from the PDB structures using the Debye equation employing the same atomic Gaussian form factor with σ=1.0 Å for all atoms (only non-H atoms included). A hydration layer using dummy atoms placed close to the protein surface is added to account for the well-defined layer of water molecules at the surface of the protein. The program uses an average atom for non-H atoms with an average mass, number of electrons, and volume corresponding to an excess scattering length per mass of Δ*ρ_m_* = 2.0 x 10^10^ cm/g. Thus, the SAXS curve is on absolute scale so that the protein concentration, which scales the intensity at *q* = 0, is a fit parameter that can be compared to the concentration determined by other methods, e.g. absorbance at 280 nm.

D2 symmetry was applied to both native A2M and A2M-MA, as well as the A2M subunits of A2M-T. Our rigid-body refinement program allows the symmetry-related molecules to be translated and rotated. The Cα atoms’ positions are used for calculating a measure of overlap and adding restraints between the molecules/domains. The overlap measure is 1/*r*^4^ weighted by the (partial) distance distribution function between pairs of Cα atoms in different subunits, where *r* is the distance between them. The distance term is a parabolic function (*r* − *r*_0_)^2^, where the restraint *r*_0_ has a target value of 6.0 Å for disulfide bridges and 3.8 Å for Cα atoms along the polypeptide backbone, and *r* is the distance between pair of Cα atoms. Distance restraints with these target values were added to the functional at all positions where the backbone was broken by the separation into rigid bodies (except at the bait region of A2M-T, where a string of residues was removed) and for the disulfides linking the disulfide-bridged dimers of A2M-MA and A2M-T, but not for native A2M where these disulfides were not modelled. The overlap measure and distance restraints are added to χ^2^ with weights that were determined by performing initial tests runs, so that they influence the structural solution, but still allow good fits to the SAXS data. A random search is used for optimizing the functional. All angles and positions are moved simultaneously for one domain at a time in cycles. Runs with the monomer as one rigid body were performed using only distance restraints for intra-dimer disulfide bridges. However, this did not lead to sufficient improvement of χ^2^ and therefore the structures were further subdivided into smaller rigid bodies.

The rigid body definitions are given in Table S1. A2M-MA and A2M-T were divided into 8 bodies per subunit, with the majority of the bait region (residues 690-719) removed from A2M-T to account for its cleavage by trypsin. For A2M-T, two bovine trypsin proteases were added using the 1F0T structure (70). Symmetry was not applied to the two trypsin proteases, which were initially placed at ± 25 Å relative to the center of the starting model. Native A2M was divided into 10 bodies per subunit. To obtain good fits for native A2M, the weight of the excluded volume measure and the distance restraints were set ten times lower than for the rigid-body refinement of A2M-MA and A2M-T. Additional inter-subunits restraints were introduced for native A2M refinement, as shown in Table S5.

D2 symmetry of the A2M tetramer was maintained during rigid body refinement. A total of 50 optimizations cycles were performed with starting maximum translation amplitudes of ± 7.5 Å and maximum rotations of ± 0.05 radians (± 1.43 °) which were both halved four times during the cycles. Subsequently, 400 cycles were performed with maximum translations of ± 0.5 Å and maximum rotations of ± 0.01 radian (± 0.286 °). For the A2M-T samples, the amplitudes of the rotations were 10 times larger for the trypsin proteases than for A2M bodies throughout the optimizations, as the orientations of the trypsins were unknown.

### TABLE S1. **SAXS information**

| Description | Human A2M (plasma-purified)  Pancreatic bovine trypsin (Sigma-Aldrich) |
| --- | --- |
| Extinction coefficient ε (wavelength and units) | 280 nm, 145440 M^-1^ cm^-1^ per subunit |
| Molecular mass *M* from chemical composition (Da) | 160809, per subunit, without glycans |
| Concentration | 1.0-1.6 mg mL^-1^, calculated from absorbance at 280 nm. |
| Solvent composition and source | HBS (aqueous 20 mM HEPES, 150 mM NaCl, pH 7.4) |
| Source, instrument and description or reference | Excillum Metal Jet Source, Bruker AXS NanoSTAR with homebuilt scatterless slits |
| Wavelength (Å) | 1.34 |
| Beam geometry (size, sample-to-detector distance) | 1.5 mm Ø, 0.90 m |
| *q*-measurement range (Å^−1^) | 0.010-0.415 |
| Absolute scaling method | Scattering from pure water at 20 ^o^C |
|  |  |
| Exposure time, number of exposures | 1800 s, one exposure per sample |
| Sample temperature (ºC) | 20 °C |
| SAXS data reduction | Logarithmic rebinning done using the *SUPERSAXS* package (CLP Oliveira and JS Pedersen, unpublished) |
| Basic analysis: Guinier, *p(r)* | In-house program for least-squares and IFT analyses (39) |
| Atomic structure modelling method | Rigid-body refinement using an in-house program (68, 69) |
| Atomic structure modelling | Models based on PDB entries 4ACQ and 1F0T |
| Modelling of missing sequences from PDB files | Manual construction using PyMOL and Coot |
| Molecular graphics | *PyMOL version 2.3.1* |
| Atomic structure modelling program | *WLSQ_SYMXV6XLD, WLSQ_SYMXV6XLDX, WLSQ_SYMXV6XL_TRYPDX* |
| Constant background varied | Yes |
| *q* range for fitting [Å^-1^] | 0.014-0.415 |
| Symmetry | D2 for A2M, no symmetry for trypsins in A2M-T |
| Mean-square residual value | 1.3-3.7 |

### TABLE S2. **Rigid-body definitions used in model refinement against SAXS data.**

| A2M conformation | Rigid bodies |
| --- | --- |
| Native A2M | **10 bodies for each A2M monomer:**  MG1(24-126)+LNK(663-685)  MG2(127-224)  MG3(225-345)  MG4(346-450)  MG5(451-564)  MG6(564-601,737-790)+LNK(602-625)+BR(729-737)  MG7(791-910)  CUB(911-952,1273-1334)  TE(953-1272)  RB(1335-1474)  Note that the flexible LNK loop (residues 626-662) and the bait region (residues 686-728) were not included in native A2M. |
| A2M-MA | **8 bodies for each A2M monomer:**  MG1(24-126)+ MG5(451-564) +LNK(625-689)+BR(690-709)  MG2(127-224)+ MG6(564-601,737-790)+LNK(602-624)+BR(710-737)  MG3(225-345)  MG4(346-450)  MG7(791-910)  CUB(911-952,1273-1334)  TE(953-1272)  RB(1335-1474) |
| A2M-T | **8 bodies for each A2M monomer, 1 body for each trypsin:**  A2M-T used the same bodies as for A2M-MA, except residues 690-719 were removed from the bait region due to cleavage. Residues 690-704 are therefore expected to be mobile; in order to avoid steric clashes, these were also removed. Two trypsin proteases (PDB 1F0T) (70) were placed inside the hollow core of the A2M-MA-based model. |

TABLE S3: **Guinier fits and IFT results for the forward scattering, and Guinier radii**.

|  | Guinier |  |  |  | IFT |  |  |  | | Theoretical |
| --- | --- | --- | --- | --- | --- | --- | --- | --- | --- | --- |
|  | *I*(0) [cm-1] | *R_g_* [Å] | Mass (kDa) |  | *I*(0) [cm-1] | *Rg* [Å] | Mass (kDa) | |  | Mass (kDa) |
| **Glycosylated** |  |  |  |  |  |  |  | |  |  |
| A2M-MA | 0.780 ± 0.002 | 67.3 ± 0.1 | 695 |  | 0.765 ± 0.002 | 65.5 ± 0.1 | 681 | |  | 720 |
| Native A2M | 0.646 ± 0.005 | 76.7 ± 0.4 | 700 |  | 0.632 ± 0.002 | 74.1 ± 0.2 | 685 | |  | 720 |
| A2M-T | 0.462 ± 0.004 | 66.4 ± 0.3 | 722 |  | 0.456 ± 0.001 | 65.1 ± 0.2 | 722 | |  | 766 |
| **Deglycosylated** | |  |  |  |  |  |  | |  |  |
| A2M-MA | 0.500 ± 0.003 | 65.6 ± 0.3 | 622 |  | 0.494 ± 0.001 | 64.2 ± 0.1 | 615 | |  | 643 |
| Native A2M | 0.548 ± 0.004 | 76.1 ± 0.3 | 630 |  | 0.535 ± 0.001 | 73.4 ± 0.1 | 615 | |  | 643 |
| A2M-T | 0.808 ± 0.004 | 65.9 ± 0.3 | 665 |  | 0.799 ± 0.002 | 65.1 ± 0.1 | 657 | |  | 689 |

### TABLE S4. **Concentrations of samples determined from absorbance at 280 nm and SAXS modelling**.

|  | Calculated from A280 (mg/mL) | Modelling (mg/mL) |
| --- | --- | --- |
| Glycosylated: |  |  |
| A2M-MA | 1.5 | 1.69 |
| Native A2M | 1.6 | 1.46 |
| A2M-T | 1.1 | 0.96 |
| Deglycosylated: |  |  |
| A2M-MA | 1.3 | 1.21 |
| Native A2M | 1.3 | 1.36 |
| A2M-T | 1.6 | 1.83 |

### TABLE S5. **Values of the considered distance restraints in the rigid-body refinement to SAXS data**. The lengths of the intra-dimer disulfide C_α_ distances and inter-body C_α_ distances (which reflects peptide backbone continuity across rigid body divisions) are given together with their target values. The error given after the inter-body C_α_ distances is the standard deviation of the distribution of values.

| Sample | Intra-dimer  disulfide C_α_ dist. (Å) | Target  value (Å) |  | Inter-body  C_α_ dist. (Å) | Target  value (Å) |
| --- | --- | --- | --- | --- | --- |
| **Glycosylated** |  |  |  |  |  |
| A2M-MA | 5.87 | 6.00 |  | 4.02±0.42 | 3.80 |
| Native A2M | - | - |  | 9.6±4.0 | 3.80 |
| A2M-T | 6.07 | 6.00 |  | 3.92±0.18 | 3.80 |
| **Deglycosylated** |  |  |  |  |  |
| A2M-MA | 5.95 | 6.00 |  | 4.21±0.40 | 3.80 |
| Native A2M | - | - |  | 10.3±5.0 | 3.80 |
| A2M-T | 6.03 | 6.00 |  | 3.76±0.19 | 3.80 |

TABLE S6. **Additional restraints used for rigid-body refinement of native A2M SAXS models.** In order to bias the native A2M models derived from rigid-body refinement to the SAXS data towards the structure of the tetramer as seen in EM, seven additional inter-subunit restraints were introduced. Target values were taken from the native A2M EM model, and the values are from the most representative models. Restraints involving two different residue positions were included twice per subunit pair (i.e. restraint #1, Lys1047/Val899, was included both as chain A Lys1047 / chain D Val899 and chain A Val899 / chain D Lys1047).

**Glycosylated native A2M**

|  | Subunit pair | Residue 1 | Residue 2 | Target Value (Å) | Value (Å) | ∆ (Å) |
| --- | --- | --- | --- | --- | --- | --- |
| 1 | Vicinal | LYS 1047 | VAL 899 | 107.70 | 105.97 | 1.73 |
| 2 | Opposite | PHE 669 | PHE 669 | 82.00 | 82.67 | 0.67 |
| 3 | Opposite | PHE 669 | LYS 1047 | 67.30 | 64.18 | 3.12 |
| 4 | Disulfide | SER 273 | LYS 426 | 40.00 | 42.59 | 2.59 |
| 5 | Disulfide | PHE 286 | GLU 440 | 49.00 | 51.24 | 2.24 |
| 6 | Disulfide | SER 273 | GLU 440 | 37.00 | 39.15 | 2.15 |
| 7 | Disulfide | PHE 286 | LYS 426 | 52.00 | 54.61 | 2.61 |

**Deglycosylated native A2M**

|  | Subunit pair | Residue 1 | Residue 2 | Target Value (Å) | Value (Å) | ∆ (Å) |
| --- | --- | --- | --- | --- | --- | --- |
| 1 | Vicinal | LYS 1047 | VAL 899 | 107.70 | 106.07 | 1.63 |
| 2 | Opposite | PHE 669 | PHE 669 | 82.00 | 82.40 | 0.40 |
| 3 | Opposite | PHE 669 | LYS 1047 | 67.30 | 63.99 | 3.31 |
| 4 | Disulfide | SER 273 | LYS 426 | 40.00 | 41.97 | 1.97 |
| 5 | Disulfide | PHE 286 | GLU 440 | 49.00 | 51.91 | 2.91 |
| 6 | Disulfide | SER 273 | GLU 440 | 37.00 | 38.84 | 1.84 |
| 7 | Disulfide | PHE 286 | LYS 426 | 52.00 | 54.91 | 2.91 |

###
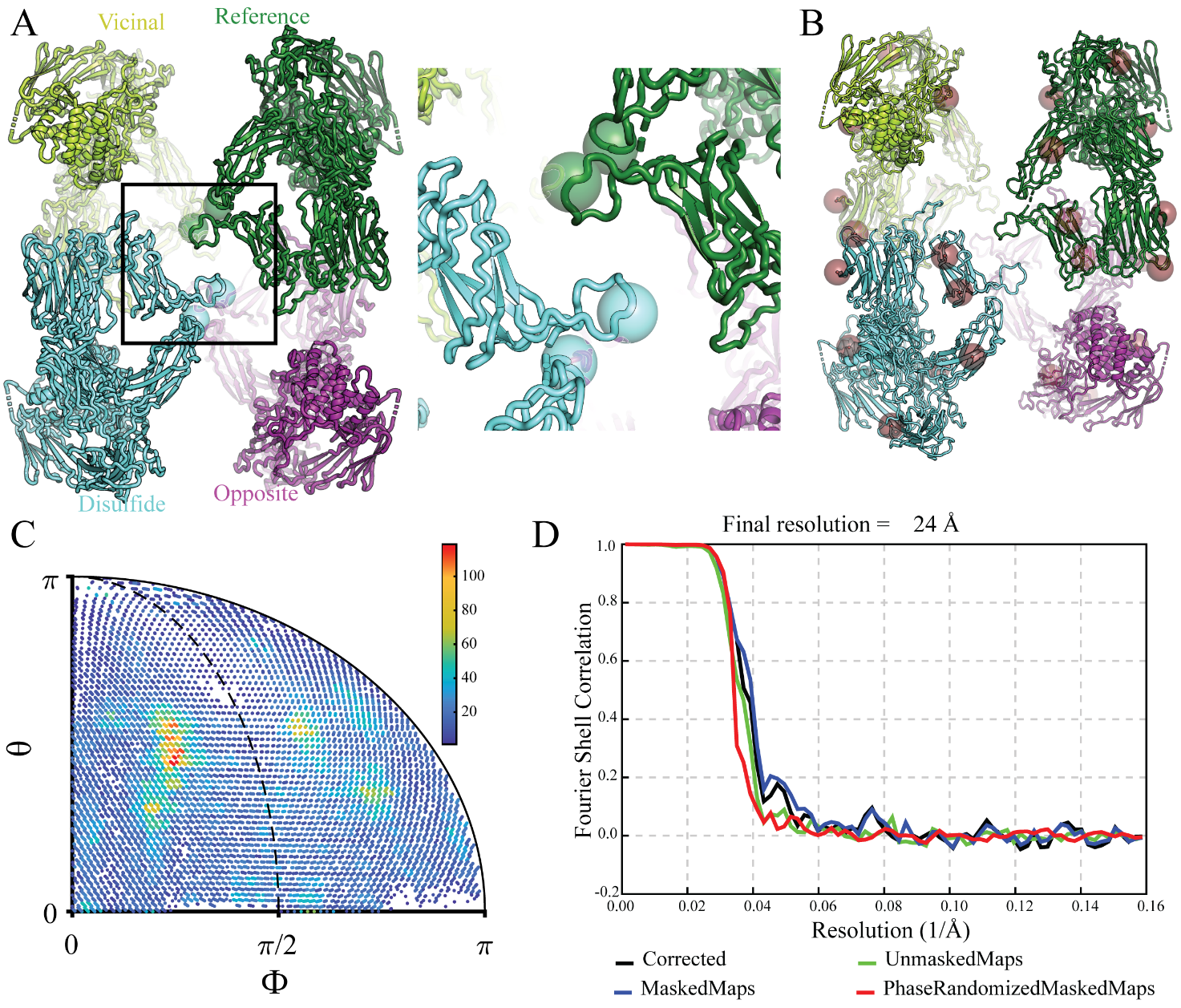


### FIGURE S1. **Fitting the native A2M subunit model into the EM 3D reconstruction.** (**A**) The fit A2M tetramer model accounts for the inter-subunit disulfide bridges between residues Cys278 and Cys431, magnified in inset. Spheres mark the C_α_ atoms of the cysteines forming the disulfides. (**B**) Visualization of the glycan distribution on A2M. Red spheres mark the positions of the C_α_ atoms of the asparagine residues carrying the Asn-linked glycans. (**C**) The refined angular distribution of the particles after 3D refinement in RELION, where the Euler angles Φ and Θ are plotted against each other using a Mollweide projection. The number of particles at each position is indicated as a heatmap as described by the adjacent color bar going from blue (few) to red (many). (**D**) The corrected, masked, unmasked and phase randomized Fourier shell correlation as a function of resolution (Å^-1^).


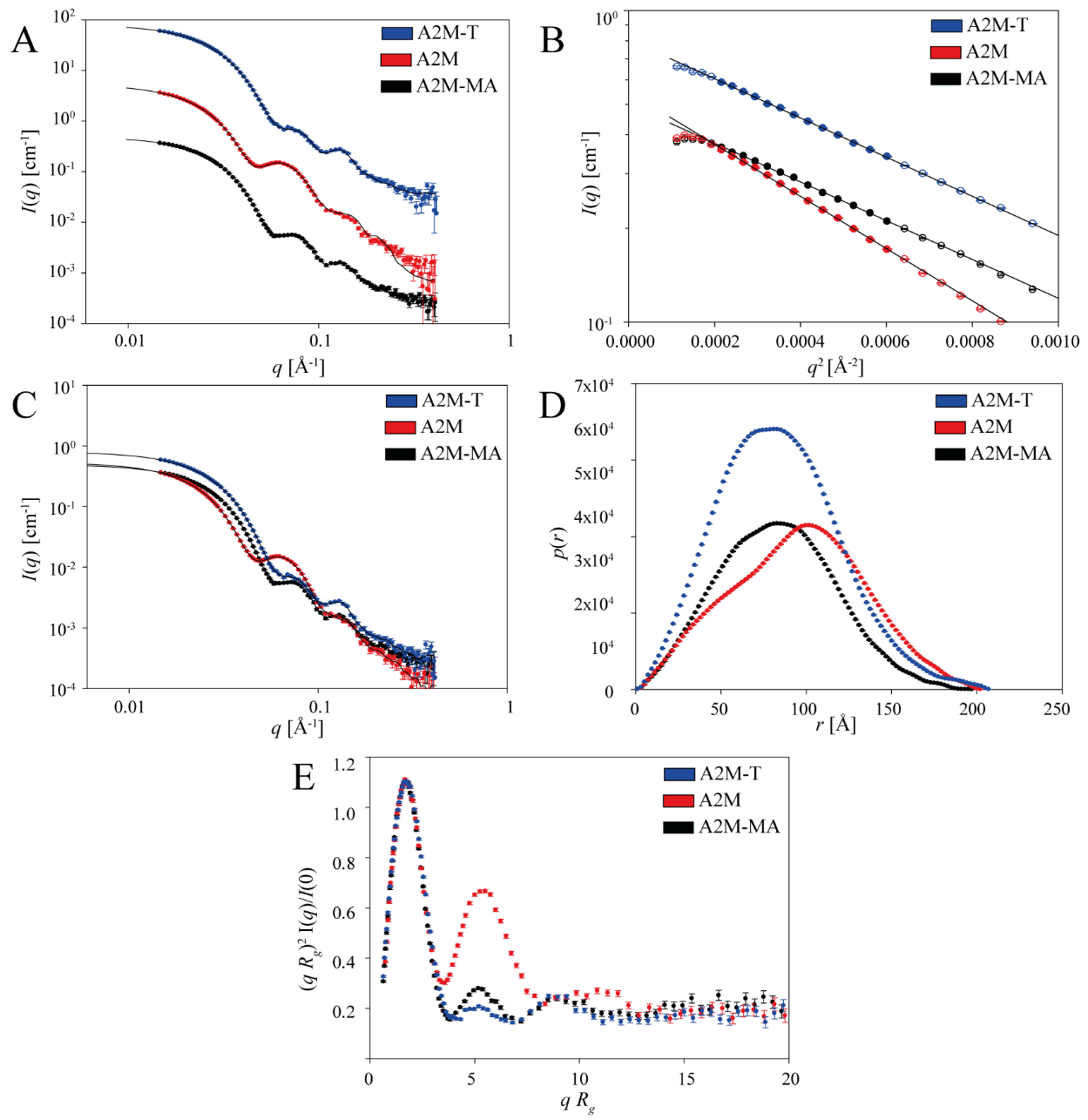


FIGURE S2. **Presentation and analysis of SAXS data from the deglycosylated A2M samples.** (**A**) Measured SAXS data and predicted SAXS curves from the most representative models obtained from rigid-body refinement (shown in Fig. S3). A2M and A2M-T data are offset 10-fold and 100-fold, respectively. (**B**) Guinier plots and fits. Only data points with full symbols are included in the fits. (**C**) SAXS data and indirect Fourier transformation fits. (**D**) Pair distribution functions from indirect Fourier transformation, normalized to I(0). (**E**) Dimensionless Kratky plot of the SAXS data.


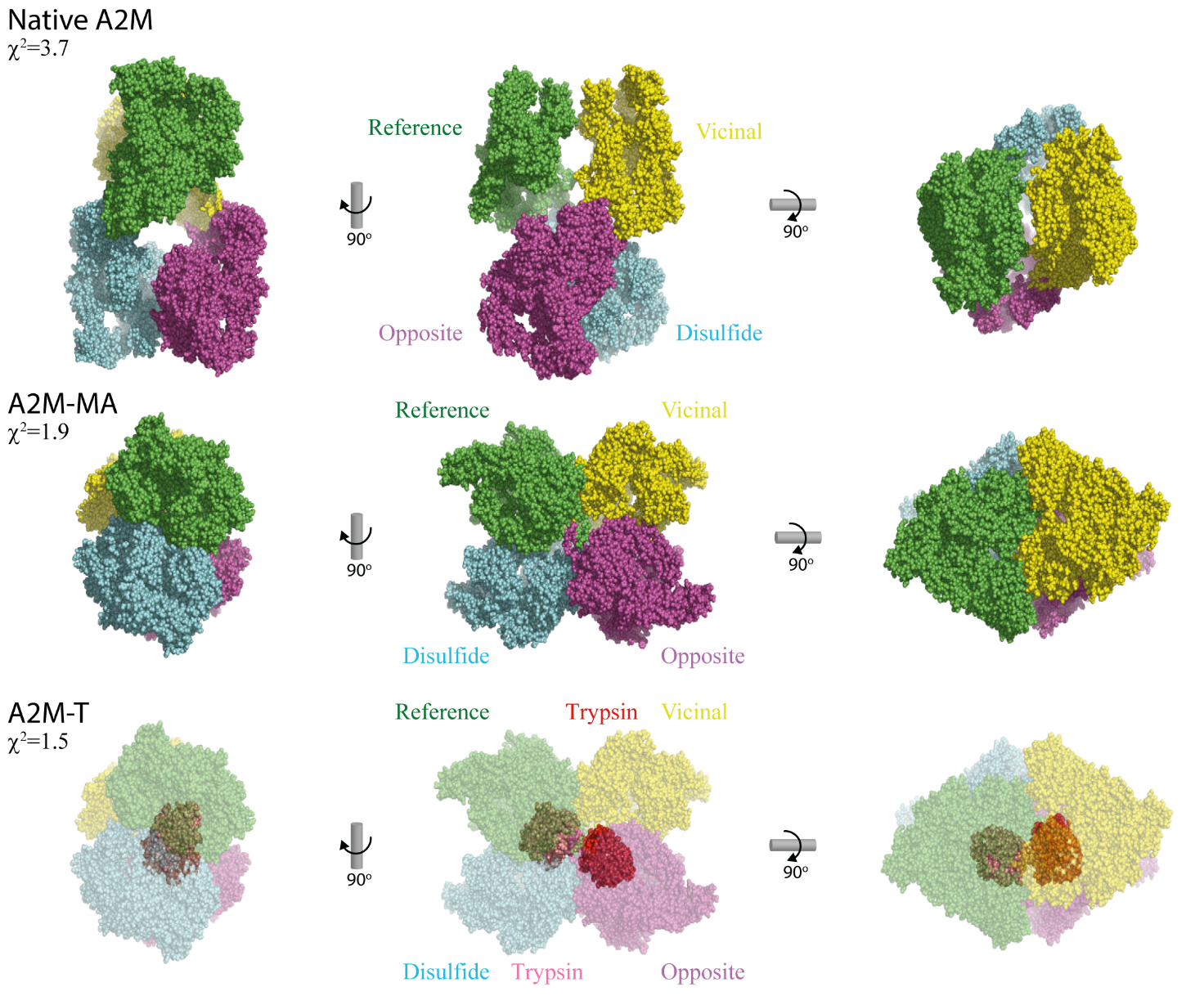


### FIGURE S3 – **SAXS-derived models of deglycosylated A2M**. Rigid-body refinement was used to optimize models against the SAXS data collected from deglycosylated native A2M, A2M-MA, and A2M-T. In each case, the most representative models are shown in three different orientations, to the same scale. The A2M subunits are transparent in A2M-T to make the internal trypsins visible; the A2M-T tetramer itself is very similar to that of A2M-MA. The deglycosylated models are overall very similar to the glycosylated models presented in Figure 4.


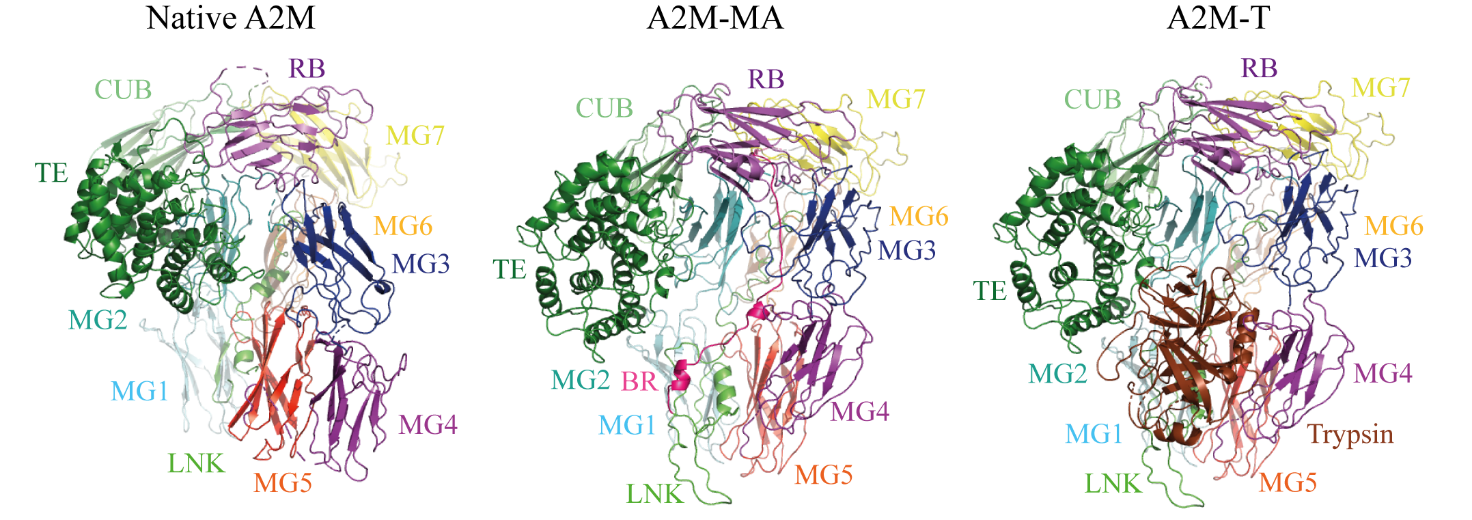
FIGURE S4. **SAXS-derived models of A2M subunits**. The subunits of the SAXS-derived A2M models (from glycosylated samples, as seen in Figure 4) are shown, colored by individual domains. The MG rings (MG1-6) of the three conformations are similar. In native A2M, the TE domain is positioned above the MG ring, where it contacts the MG2 domain, whereas in A2M-MA and A2M-T, the TE domain has moved towards the base of the MG ring and is now close to both the MG1 and MG2 domains. Due to the TE domain migration, the CUB domain is positioned closer to the MG ring in A2M-MA and A2M-T compared to native A2M. This conformational change is similar to those occurring in C3 and C4 upon proteolytic activation, although their TE domains migrate to the base of the MG ring next to the MG1 domain. Note that the bait region was not modelled in native A2M, and is mostly removed by trypsin cleavage in A2M-T.


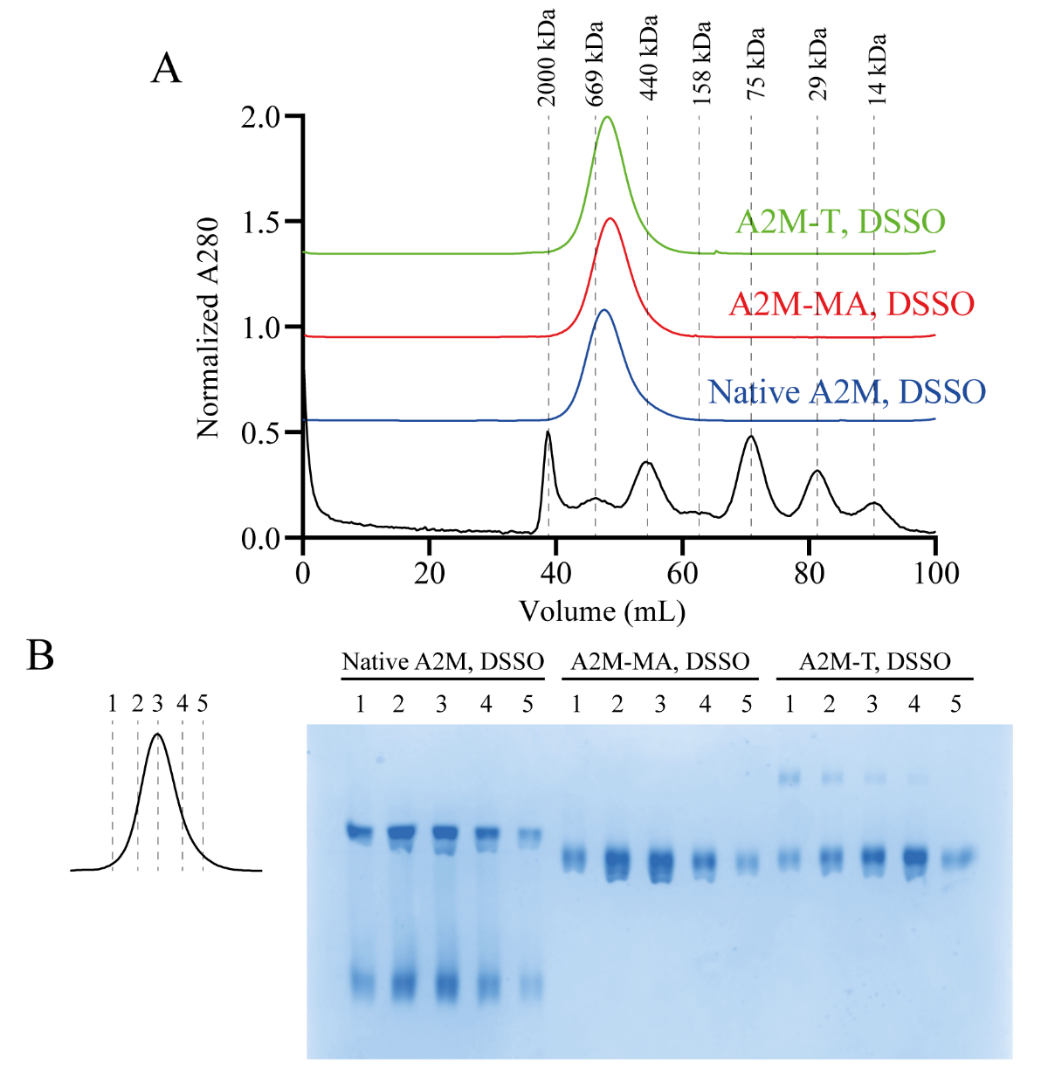


FIGURE S5. **Size exclusion chromatography of DSSO-cross-linked A2M.** (**A**) A2M in three conformations was cross-linked using DSSO. Size exclusion chromatography (SEC) on a Sephacryl S-300 HR column with HBS as running buffer was then used to isolate A2M tetramers and remove cross-linker reagents. Cross-linked A2M migrated as a broad peak in all three conformations. Although tetrameric A2M (720 kDa) is not well resolved from larger species, A2M dimers (360 kDa) can be resolved and were not detected. The black curve shows calibration of the SEC column with size markers from 14 to 2000 kDa. (**B**) Pore limited native PAGE was used to analyze fractions from the beginning, middle, and end of the elution peak from each cross-linked A2M’s SEC purification, as indicated on the chromatogram. Cross-linked native A2M, which had been observed to dissociate into dimers prior to SEC, showed similar tetramer:dimer ratios in fractions across its elution peak, indicating that dissociation to dimers was an artifact of native PAGE and that the sample was tetrameric in solution. A2M-MA was entirely tetrameric across its elution peak. A2M-T formed a small amount of octamer-sized cross-linked product which was most abundant at the beginning of its SEC elution peak. Fractions towards the tail of its peak were therefore used for further XL-MS analysis to prevent the detection of inter-tetramer cross-links.


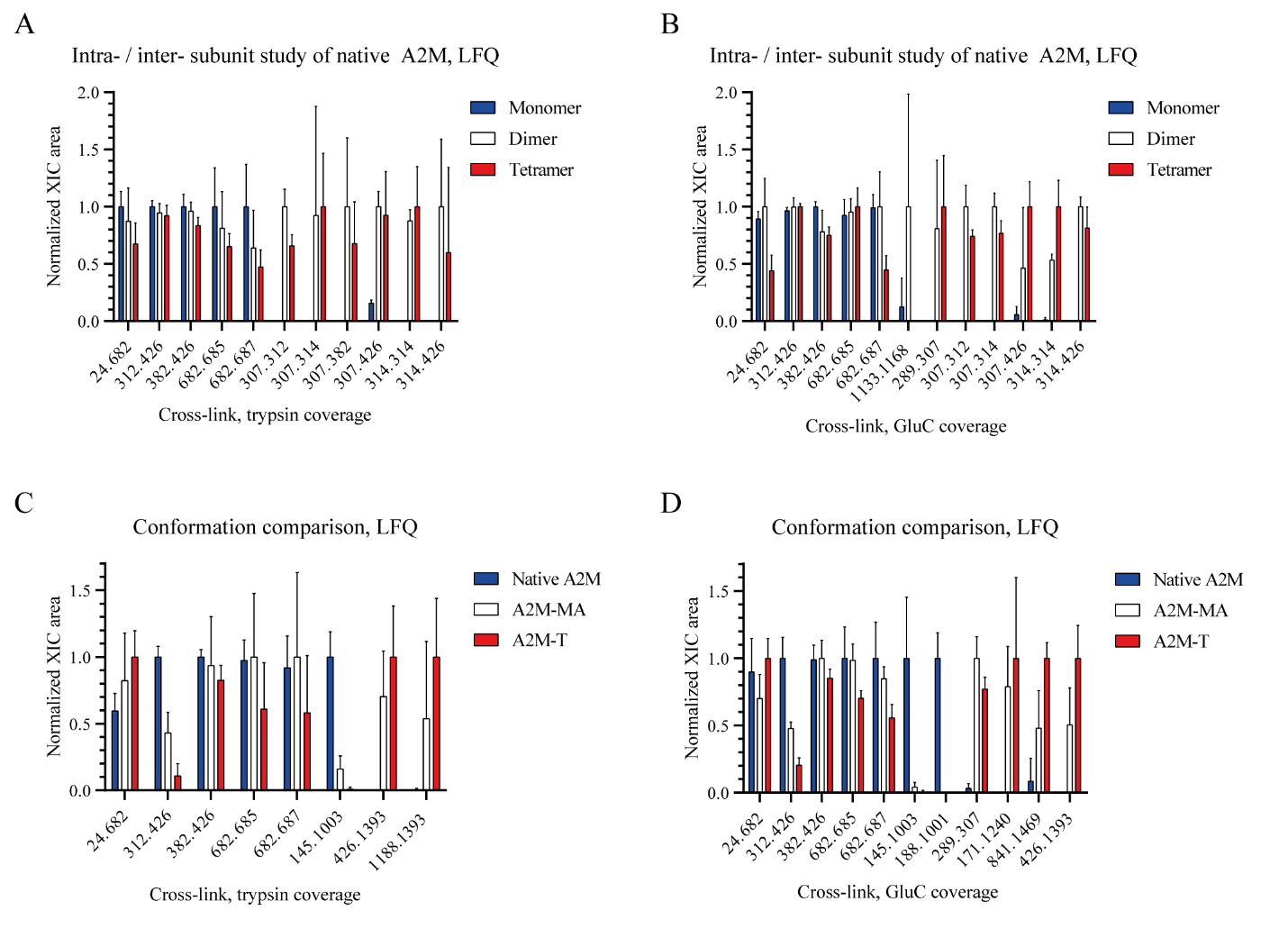
FIGURE S6. **Label-free quantification of A2M cross-links.** Cross-linked peptides were quantified based on their extracted ion count (XIC) areas at the MS1 level. XIC abundances were normalized to the sample in which they were highest. Five intra-subunit cross-links (the first five from the left) covering the relatively static MG ring are included in all four quantifications for comparative purposes. For all panels, *n* = 5 (based on five technical replicates of each peptide sample) and error bars give the standard deviation. Samples digested with trypsin (**A** and **C**) or with both trypsin and GluC (**B** and **D**) were quantified using Proteome Discoverer 2.4; samples digested with both chymotrypsin and trypsin were not quantified. (**A** and **B**) The monomer, dimer, and tetramer SDS-PAGE bands of native A2M were analyzed by XL-MS to identify intra- and inter-subunit cross-links. Ten cross-links were found in the dimer and not in the monomer, and label-free quantification was used in addition to identification to evaluate whether cross-links were dimer-specific and therefore inter-subunit. 307.312, 307.314, 307.382, 314.314, 314.426, and 289.307 were determined to be dimer-specific. 307.426 was present in the monomer band, but at low abundance compared to the dimer and tetramer samples, and therefore likely represents a cross-link that is both intra- and inter-subunit. (**C** and **D**) Total protein digests of cross-linked A2M in its three conformations were analyzed by XL-MS to elucidate the structures of each conformation. Seven cross-links were found that were identified in both native A2M and collapsed A2M (A2M-MA and A2M-T) but only consistent with one of the A2M models. These cross-links were quantified to determine whether they could be due to contamination. 145.1003 and 188.1001 were found to be at least 10-fold more abundant in native A2M, and their detection in A2M-MA and A2M-T was considered to be due to incomplete conversion of native A2M to the respective activated conformations. Likewise, 426.1393, 1188.1393, 289.307, 171.1240, and 841.1469 were at least 10-fold more abundant in A2M-MA and A2M-T than in native A2M, and their detection in native A2M was considered to be due to traces of protease-cleaved A2M in the plasma-purified A2M preparation.


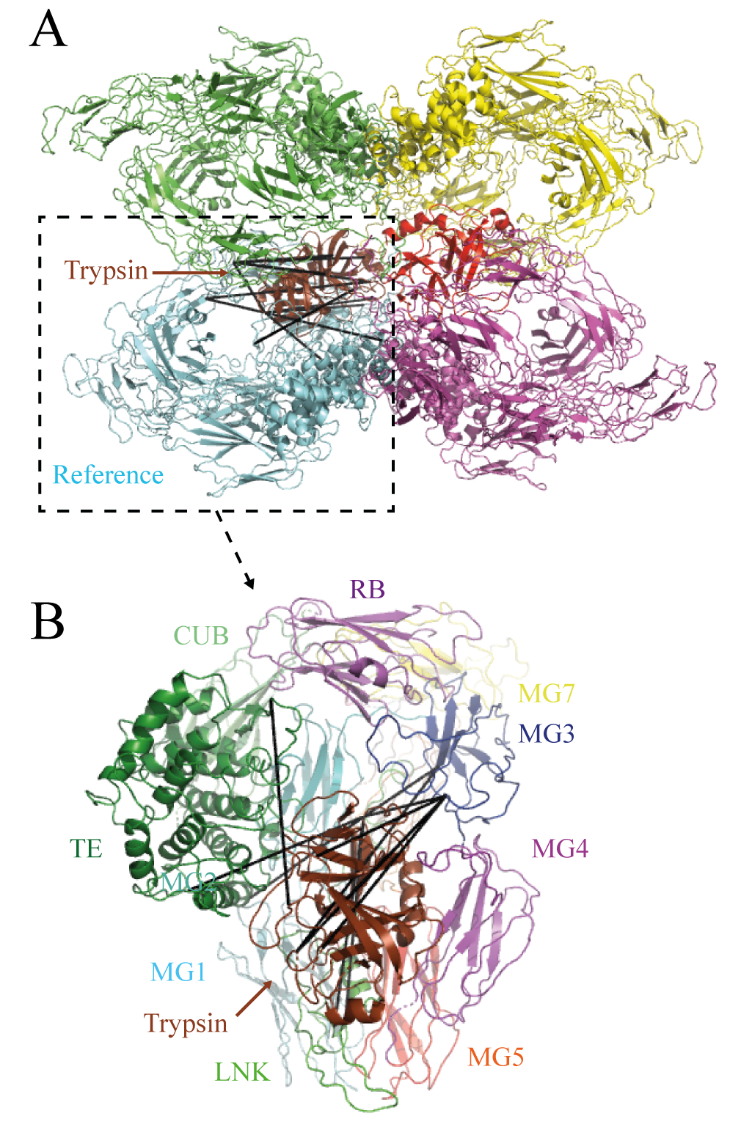


FIGURE S7. **Cross-links between A2M and trypsin.** All DSSO cross-links between trypsin and A2M identified in A2M-T are shown in the A2M tetramer (**A**) and an isolated trypsin with its closest A2M subunit (**B**). Cross-links to the inner face of the A2M subunit were identified, supporting the SAXS-identified position of trypsin within the hollow interior of A2M-T. The identified cross-links are not compatible with a single fixed trypsin position or orientation, indicating that trypsin is heterogeneously trapped. The cross-link positions are given in Supplementary Spreadsheet 1.


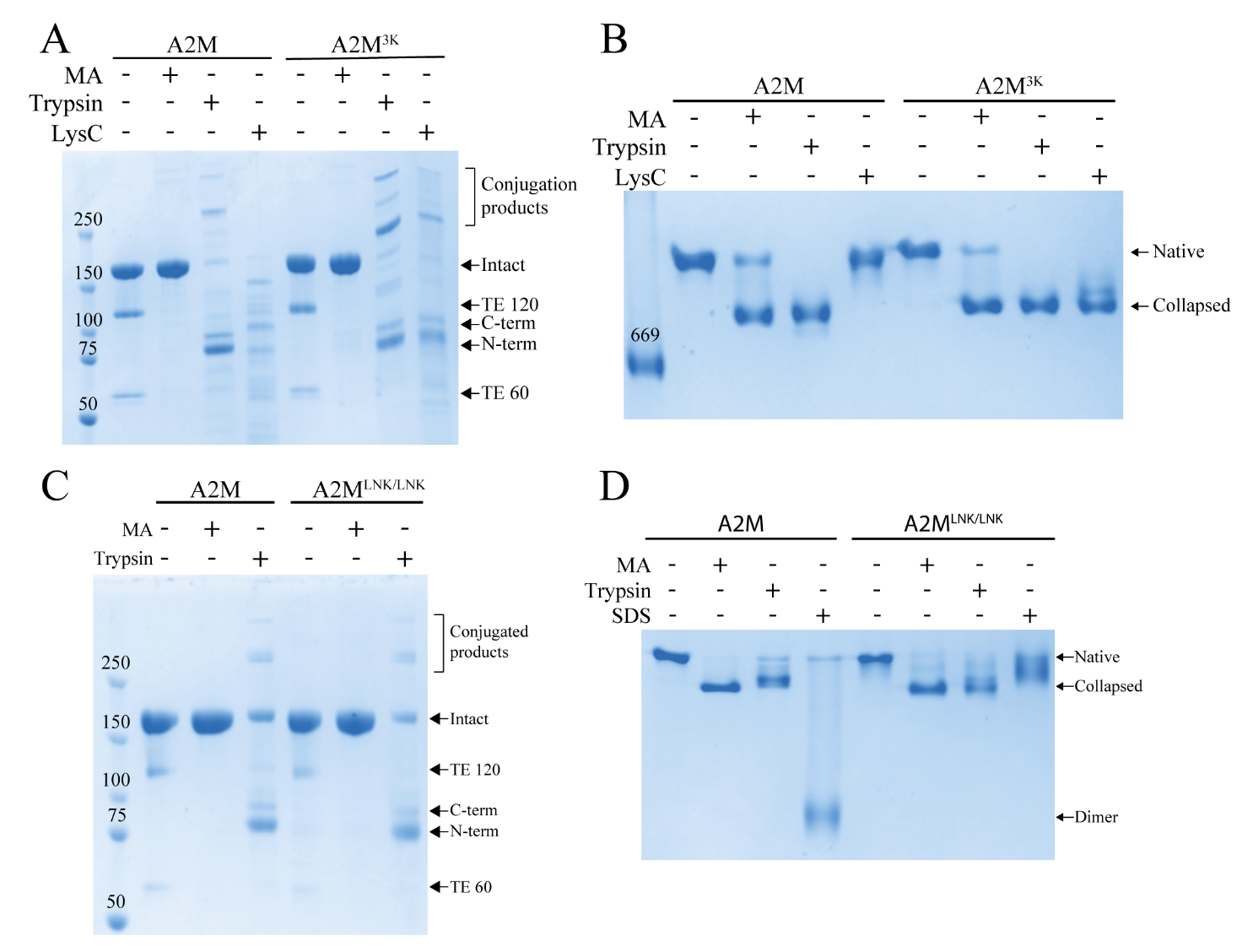


FIGURE S8. **Basic functionality of the A2M^3K^ and A2M^LNK/LNK^ mutants.** (**A, C**) Reducing SDS-PAGE and (**B, D**) pore limited native PAGE of plasma-purified A2M and recombinant A2M^3K^ (**A, B**) or A2M^LNK/LNK^ (**C, D**), when native or treated by methylamine or trypsin under identical conditions; the lysine-specific protease LysC is also used in the experiments with A2M^3K^. The formation of high MW conjugation products between A2M and protease is indicative of thiol ester-mediated protease conjugation. A conformational collapse is apparent under native conditions for all A2Ms when treated by methylamine or trypsin, but only A2M^3K^ collapses when cleaved by LysC. Both A2M mutants retain the native conformation, thiol ester, and protease- or methylamine-induced conformational collapse.


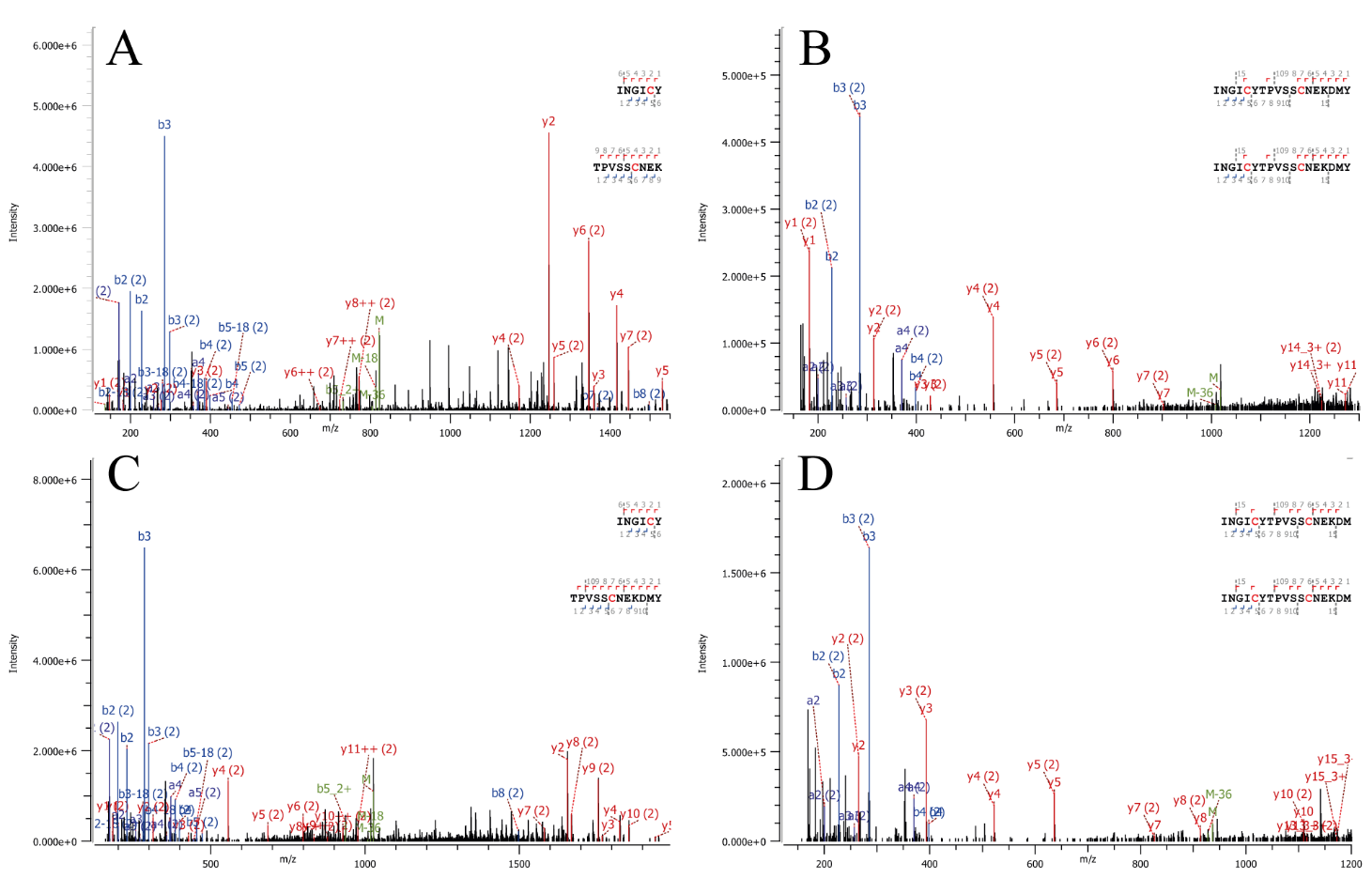


FIGURE S9. **Additional MS2 spectra of the novel A2M^LNK/LNK^ disulfide.** Methylamine-treated, carbamidomethylated A2M^LNK/LNK^ (without reduction) was digested at pH 6 with trypsin, chymotrypsin, or both trypsin and chymotrypsin, or at pH 3 with pepsin. Low pH digests were used to prevent disulfide scrambling under denaturing conditions. Peptide spectral matches covering the novel Cys654 and Cys661 found using Byonic (Protein Metrics) are shown (with *y* fragment ions in red and *b* fragment ions in blue); trypsin alone did not cover the cysteines, trypsin+chymotrypsin gave spectrum **A**, chymotrypsin alone gave spectra **B** and **C**, and pepsin gave spectrum **D**. While spectra **A** and **B** demonstrate disulfide formation between Cys654 and Cys661, they do not exclude the possibility of intra-subunit disulfide formation. In contrast, spectra **C** and **D** do not distinguish between two 654/661 disulfides or a 654/654 and a 661/661 disulfide, but they demonstrate that the disulfides are inter-subunit.


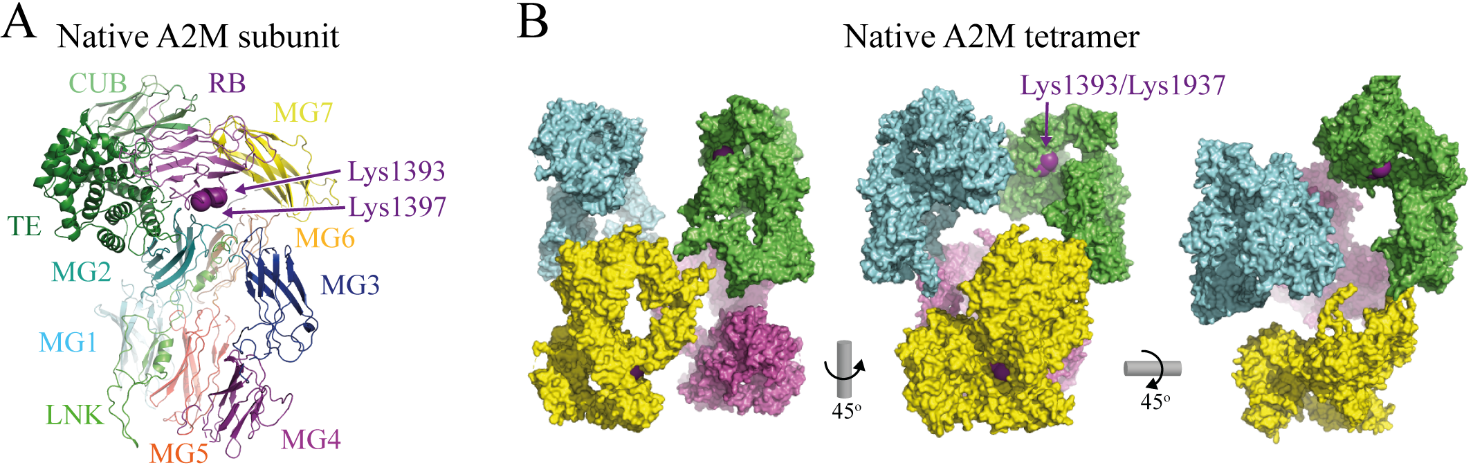
FIGURE S10. **Position of the receptor-binding site in native A2M.** The positive of Lys1393 and Lys1397, which are required for LRP1 and Grp78 receptor binding, respectively, are shown as the indicated spheres on a cartoon representation of a native A2M subunit (**A**) and on a surface representation of the native A2M tetramer (**B**), using the model derived from fitting to the EM reconstruction. Although the receptor-binding residues are surface-exposed even in the native conformation of A2M, they are oriented towards the interiority of A2M and are inaccessible to their receptors.
